# New insights into SREB orphan receptor roles in the ovary, using comparative transcriptomics across 3 fishes

**DOI:** 10.1210/endocr/bqag089

**Published:** 2026-08-07

**Authors:** Charles F Heyder, Casey A Murray, Philip Granith, Mukhriddin Makhkamov, Emma Bourget, Isabella Cain, Breckon Davidson, Taylor-Jeffery Doone, Joanna Korasadowicz, Nora Hull, Jade Martens, Ryan Martin-Hachey, Keira McGrath, Haley Stewart, Jamoliddin Razzokov, Parthiban Marimuthu, Thanigaimalai Pillaiyar, Julien Hanson, Christopher J Martyniuk, Matthew A DiMaggio, Timothy S Breton

**Affiliations:** Tropical Aquaculture Laboratory, Program in Fisheries and Aquatic Sciences, School of Forest, Fisheries, and Geomatics Sciences, Institute of Food and Agricultural Sciences, University of Florida, Ruskin, FL 33570, USA; Tropical Aquaculture Laboratory, Program in Fisheries and Aquatic Sciences, School of Forest, Fisheries, and Geomatics Sciences, Institute of Food and Agricultural Sciences, University of Florida, Ruskin, FL 33570, USA; Faculty of Science and Engineering, Pharmaceutical Science Laboratory (PSL–Pharmacy) and Structural Bioinformatics Laboratory (SBL–Biochemistry), Åbo Akademi University, Turku FI-20500, Finland; Faculty of Science and Engineering, Pharmaceutical Science Laboratory (PSL–Pharmacy) and Structural Bioinformatics Laboratory (SBL–Biochemistry), Åbo Akademi University, Turku FI-20500, Finland; Institute for Advanced Studies, New Uzbekistan University, Tashkent 100000, Uzbekistan; Biology Department, University of Maine at Farmington, Farmington, ME 04938, USA; Biology Department, University of Maine at Farmington, Farmington, ME 04938, USA; Biology Department, University of Maine at Farmington, Farmington, ME 04938, USA; Department of Biological Sciences, University of Southern Maine, Portland, ME 04104, USA; Biology Department, University of Maine at Farmington, Farmington, ME 04938, USA; Biology Department, University of Maine at Farmington, Farmington, ME 04938, USA; Biology Department, University of Maine at Farmington, Farmington, ME 04938, USA; Biology Department, University of Maine at Farmington, Farmington, ME 04938, USA; Biology Department, University of Maine at Farmington, Farmington, ME 04938, USA; Biology Department, University of Maine at Farmington, Farmington, ME 04938, USA; Biology Department, University of Maine at Farmington, Farmington, ME 04938, USA; Institute of Fundamental and Applied Research, National Research University TIIAME, Tashkent 100000, Uzbekistan; Department of Natural Sciences, Karshi State Technical University, Kashkadarya 180100, Uzbekistan; Department of Biotechnology, Tashkent State Technical University, Tashkent 100095, Uzbekistan; Faculty of Science and Engineering, Pharmaceutical Science Laboratory (PSL–Pharmacy) and Structural Bioinformatics Laboratory (SBL–Biochemistry), Åbo Akademi University, Turku FI-20500, Finland; Tübingen Center for Academic Drug Discovery and Development, Eberhard Karls University Tübingen, Tübingen 72076, Germany; Laboratory of Molecular Pharmacology, GIGA Research Institute & CIRM, University of Liège, Liège 4000, Belgium; Department of Physiological Sciences, Center for Environmental and Human Toxicology, College of Veterinary Medicine, University of Florida, Gainesville, FL 32611, USA; Tropical Aquaculture Laboratory, Program in Fisheries and Aquatic Sciences, School of Forest, Fisheries, and Geomatics Sciences, Institute of Food and Agricultural Sciences, University of Florida, Ruskin, FL 33570, USA; Biology Department, University of Maine at Farmington, Farmington, ME 04938, USA

**Keywords:** gpr27, gpr85, gpr173, SREB, phoenixin, agonist

## Abstract

Superconserved receptors expressed in brain (SREB) are a family of orphan G protein–coupled receptors with 3 members in most vertebrates (GPR27 or SREB1, GPR85 or SREB2, and GPR173 or SREB3). They are associated with diverse physiological processes, ranging from glucose homeostasis to ovarian development. Despite a lack of confirmed ligands, our understanding of these receptors has increased based on interactions with putative SREB3 ligands such as phoenixin (PNX) and the development of synthetic SREB1 agonists (A8535 and PT-91). However, to date no studies have used these agonists in any complex vertebrate system. The objective of this study was to compare the *in vitro* transcriptomic responses to PNX-20, A8535, and PT-91 in the ovaries of 3 fishes with different SREB systems: (1) zebrafish (*Danio rerio*) that exhibit similar receptors to mammals, (2) mummichog (*Fundulus heteroclitus*) that lost a receptor but gained a fish-specific member, and (3) pufferfish (*Dichotomyctere nigroviridis*) that exhibit all 4 receptors. Agonist interactions with fish SREB1s were confirmed using *in silico* characterizations. Zebrafish exhibited the strongest transcriptome response, including agonist-induced changes in oxidative phosphorylation and cell adhesion pathways. Mummichog exhibited a less robust response, possibly related to receptor evolutionary divergence, while pufferfish exhibited an intermediate transcriptome response. Short-term agonist exposure did not alter ovarian steroid levels ([17]estradiol, 17-hydroxyprogesterone, cortisol, testosterone, or 11-ketotestosterone). Collectively, the data reveal previously unrecognized developmental, metabolic, neural, and vascular gene networks associated with SREB activation and suggest both receptor-specific signaling and functional overlap within this family.

## Introduction

Superconserved receptors expressed in brain (SREBs) are a family of highly conserved orphan G protein–coupled receptors with 3 members in most vertebrates: SREB1 (orphan receptor GPR27), SREB2 (GPR85), and SREB3 (GPR173) (1). SREBs were first characterized in the mammalian central nervous system but have since been studied in diverse tissues and physiological processes, ranging from neurogenesis to insulin regulation, ovarian folliculogenesis, and skeletal muscle metabolism (2-6). Among the family members, SREB1 exhibits the broadest tissue distribution and is most studied in glucose homeostasis (5, 7-9). SREB2 is expressed in neurons and is associated with schizophrenia and autism spectrum disorder (10, 11), whereas SREB3 has proposed roles in reproductive and metabolic regulation in the hypothalamus, gut, and female reproductive system (3, 4, 12-14). Our understanding of SREB3 is largely derived from the recent identification of several putative endogenous ligands, including a gonadotropin-releasing hormone metabolite (GnRH) (1-5), cholecystokinin, and a recently discovered peptide, phoenixin (PNX) (12, 15, 16).

PNX is a cleavage product of a small mitochondrial protein and has 2 major forms, PNX-14 and PNX-20, which were both implicated in reproduction by modulating the GnRH system in the hypothalamus and pituitary (17-20). PNX is also expressed in the ovary, where it stimulates estrogen production and ovulation, and is broadly associated with several disorders (4, 13, 21-23). Beyond reproduction, PNX has been linked to food intake, energy metabolism, and cardiac protection, highlighting its broader physiological significance (24, 25).

Despite these advances, the status of SREB3 as a PNX receptor remains unresolved. This proposed identification was based on a deductive ligand–receptor matching strategy, and several subsequent studies suggested that PNX actions may require functional SREB3 (19, 25, 26). However, direct pharmacological evidence remains incomplete, as ligand-binding assays have failed to conclusively verify this interaction (27). Receptor deorphanization requires binding reproducibility in at least 2 different settings, before interaction is confirmed, as a cautionary practice (28, 29). While past research demonstrates that PNX can provide insights into SREB3 functions, the receptor may act as part of a more integrative pathway requiring further research. This uncertainty is even greater for the other SREB family members, which remain orphan receptors without defined endogenous ligands.

Development of synthetic SREB agonists is challenging without known coupling to downstream mediators, but several research groups have focused on these efforts to better characterize SREB function. Initial efforts successfully developed inverse agonists (30, 31), but ligand binding was not verified and their efficacy remains unconfirmed (32-34). A major advance came from the works of Dupuis et al., who proposed that SREBs may signal through noncanonical β-arrestin-dependent mechanisms (32, 35). This group developed a chimeric model with human SREB1 and the C-terminal tail from the vasopressin V2 receptor, which binds efficiently to β-arrestin 2. Using chemical screening and confocal microscopy, selected compounds were confirmed to recruit β-arrestin 2 to the membrane of cells expressing SREB1 (32). The most potent compound was a sulfonamide derivative (termed compound I or Agonist 8535 (A8535): 2,4-dichloro-N-(4-(N-phenylsulfamoyl)phenyl)benzamide) that exhibited no cellular toxicity and was selective to SREB1. A8535 was also tested with the native human receptor, where the agonist did not activate G protein signals but did recruit β-arrestin 2 (32). Since this discovery, more potent derivatives of A8535 have been synthesized, including a compound termed 7p or PT-91 (2-methoxy-N-(4-(N-phenylsulfamoyl)phenyl)-4-(trifluoromethyl)benzamide) (33, 36). The novel PT-91 exhibited greater potency to the native SREB1 receptor (36). Neither compound has been used extensively in any animal model, with only 1 study published to date on A8535 and lactate production in rodent cells (9). These compounds have not been tested in whole-animal or comparative vertebrate systems, leaving their broader relevance unexplored.

While most information stems from research in mammals, fish have recently provided new insights into SREB functions and represent an interesting vertebrate clade with unique receptor characteristics (34, 37). Many highly derived fishes exhibit a fourth receptor that duplicated from SREB3, termed SREB3B or GPR173B, and the novel receptor was shown to exhibit divergent patterns from the mammalian homolog SREB3A (37-39). In fish, the 2 SREB3s are differentially regulated by (17) estradiol and do not respond similarly to PNX (40, 41). In addition, the 2 SREB3s exhibit divergent spatial mRNA patterns and may be differentially regulated (38, 39). Not all fishes exhibit the gene though, as less-derived fishes, such as zebrafish (*Danio rerio*), only exhibit the same 3 receptors as mammals. Some species have lost a receptor entirely, such as those of the Order Cyprinodontiformes, which lack SREB3A (37, 42). Despite these differences, research in fish has identified many conserved functions for PNX and SREBs in energy metabolism, food intake, and reproduction (42-47). Overall, the scientific community has not yet fully characterized SREB functionality. For instance, the potential for overlapping roles among receptor family members has been proposed for over a decade (10, 48). The combined use of PNX, novel SREB1 agonists, and comparative fish models with unique receptor configurations represents a promising new direction to characterize SREB function.

The objective of this study was to better elucidate conserved roles for SREBs in metabolism and reproduction. To achieve this, PNX-20, A8535, and PT-91 were used in *in vitro* ovarian exposures using 3 fish species with distinct SREB receptor complements. Zebrafish (*D. rerio*) were used because they exhibit similar receptors to mammals (SREB1, SREB2, and SREB3A). Mummichog (*Fundulus heteroclitus*) were selected because they have lost SREB3A but gained the novel receptor (SREB1, SREB2, and SREB3B) (37). Finally, green-spotted pufferfish (*Dichotomyctere nigroviridis*) were examined because they express all 4 receptors (SREB1, SREB2, SREB3A, and SREB3B). Agonist binding to fish SREB1 was first determined in all 3 species using simulated molecular dynamics (MD), followed by comparative transcriptomics and steroid quantification to characterize biological effects. We hypothesized that PNX-20 would exert different downstream effects in each species due to SREB3 divergence, while PT-91 exposure would exhibit greater responses than A8535. We also hypothesized that PNX-20 would increase steroids associated with metabolism and ovarian growth. By integrating receptor-level *in silico* analyses with comparative ovarian functional assays, this study provides the first vertebrate framework for evaluating SREB-directed agonists and offers new insight into functional specialization and overlap within this receptor family.

## Materials and methods

### MD of agonist binding to fish SREB1 receptors

MODELLER software (ver. 10.6) was used to build homology models (HMs) for zebrafish, pufferfish, and mummichog SREB1 (49) using the human GPR27 structure (36). Amino acid sequences were obtained from UniProt (The UniProt Consortium, UniProt code [UPC], 2023): zebrafish (UPC: A9JRY2), pufferfish (UPC: A0A872TNE7), and mummichog (UPC: A0A3Q2QV11). For each species, 50 models were generated, and the top quality model was selected based on visual inspection and discrete optimized protein energy (DOPE) score in MODELLER. The residue ranges of the resulting HMs are zebrafish (26-343), pufferfish (26-343), and mummichog (25-342) (Supplemental File 1, Fig. S1) (50).

**Figure 1:**
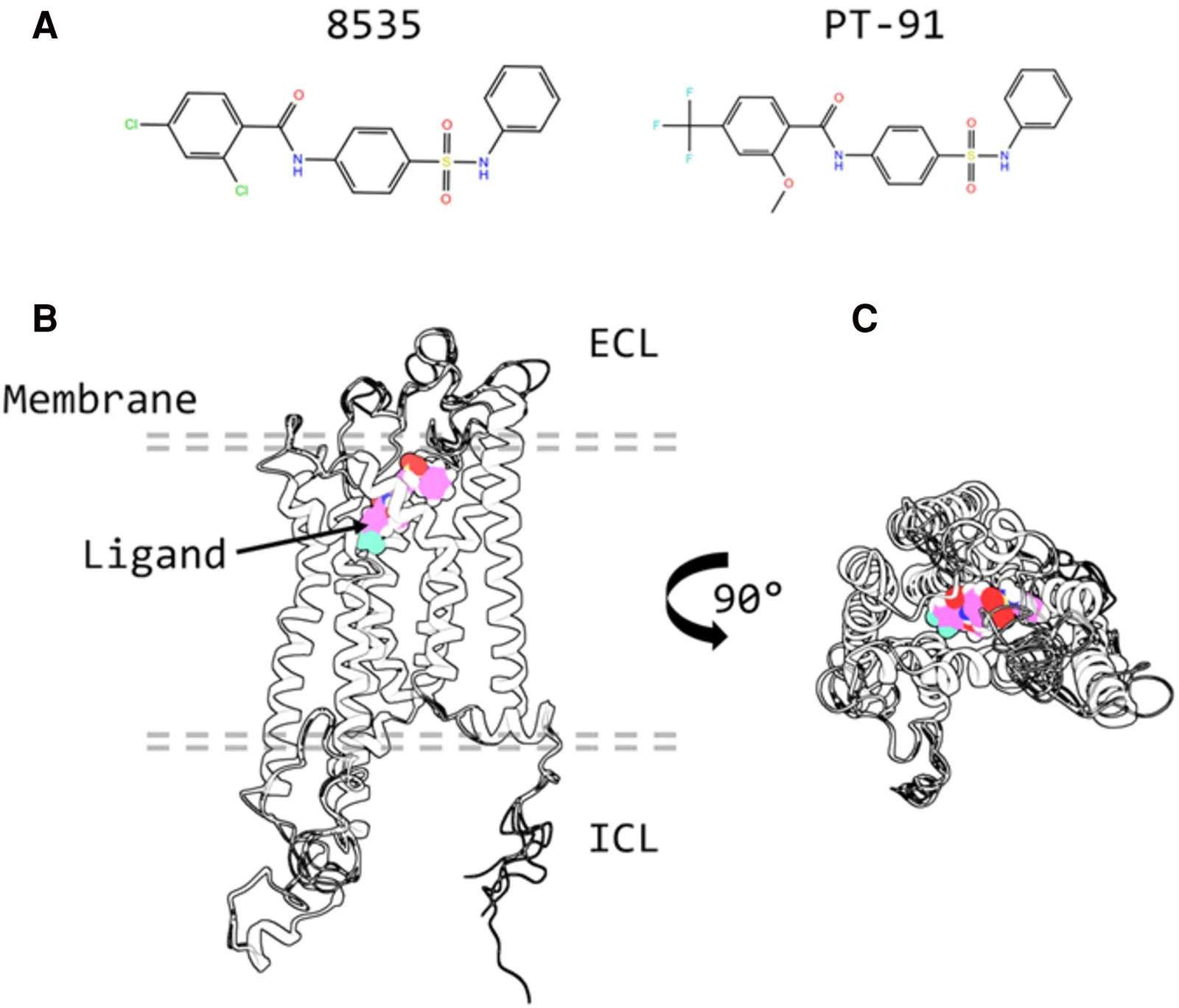
Two-dimensional representations of ligand 8535 and PT-91 (A). Side view (B) and top view (C) of HMs of fish SREB1 superposed on the human GPR27 structure. The white cartoon represents the receptors. The circles represent the PT-91 ligand in the binding pocket, with colors matching panel b and aromatic groups in purple. The gray dashed lines indicate the cell membrane from the extracellular (ECL) and intracellular (ICL) sides, respectively.

To prepare the complexes for *in silico* analysis, the models were processed using Maestro's (Schrödinger Release 2025: Maestro, Schrödinger, LLC, New York, NY, 2025) built-in tools, the Protein Preparation Workflow, which prepare the complexes by adding missing hydrogen atoms, removing water molecules, generating heteroatom states, assigning bond orders, and predicting ionizable residues (Asp, Glu, Arg, Lys, His). Protonation states at physiological pH (7.7) were predicted using the integrated PROPKA algorithm. The complexes underwent restrained energy minimization using the OPLS4 force field (51) and pH-dependent protonation states were assigned using Epik (52). The ligands (Fig. 1A) were prepared in a similar manner using LigPrep (53). Missing hydrogen atoms were added, formal charges were assigned, and stereoisomers and plausible tautomeric and ionization states were generated. Finally, ligand conformations were energy-minimized using the OPLS4 force field.

To predict the binding pose of unbound A8535, molecular docking was performed to predict its binding pose using GLIDE (54) in Schrödinger Maestro. The extra-precision mode and postdocking minimization steps were sequentially applied to refine the docked poses. The ligand coordinates from the reference structure were used to dock A8535 into the binding pocket of SREB1 HMs from the fish species. The top ranked docked pose was selected based on the degree of alignment with the reference structure ligand (36). This resulted in 3 SREB1 structures bound to PT-91 (with the ligand position retained from the reference) and A8535, with the ligand placed by docking, respectively.

To further evaluate the stability of binding poses and ligand–receptor interactions obtained from docking, the complexes were subjected to MD simulations using Desmond (55). Before simulation, the complexes were prepared in a unit cell designed to reflect a simplified cellular environment. All receptor–ligand complexes were embedded in a POPC (1-palmitoyl-2-oleoyl-sn-glycero-3-phosphocholine) lipid bilayer, representing a widely used model cell membrane, and the system was solvated with TIP3P explicit water molecules (56) in an orthorhombic simulation box. To better mimic physiological conditions, additional simulation parameters were also applied (Table S1) (50). A physiological salt concentration of 0.15 M was used, and the net charge of the system was neutralized by adding Na^+^ or Cl^−^ counterions. The OPLS4 force field was employed for all simulated atomic interactions. MD simulations were performed in the NPγT ensemble, in which the number of particles, pressure, surface tension (γ), and temperature are kept constant. This ensemble is particularly suitable for membrane systems because it allows independent fluctuations in the lateral (x–y) dimensions of the simulation box while maintaining constant surface tension, thereby minimizing artificial distortions (55).

A multistep equilibration protocol was applied. First, energy minimization was performed on the solvent and ions while restraining the solute components (protein, ligand, and membrane). This was followed by a short NVT (number of particles, volume, and temperature) simulation using Brownian dynamics at 10 K for 100 ps, with restraints on solute heavy atoms. Subsequently, a brief NPT simulation at 100 K (20 ps) was carried out with solute restraints. The system was then heated from 100 to 300 K under NPγT conditions over 10 ps, maintaining solute restraints. Restraints were gradually reduced during a series of short NVT simulations at 300 K totaling 24 ps. Finally, an unrestrained equilibration step in the NVT ensemble was performed prior to the production runs. Production runs were performed under the same conditions using the following parameters: simulation time of 3 μs, total number of frames of 2500, with a random seeded 5 replicates. Our customized Maestro tools for Simulation Quality Analysis and Simulation Interaction Diagram modules were extensively used for postsimulation analysis. The root mean square deviation (RMSD), root mean square fluctuations, trajectory frequency, and per-residue decomposition (PRD) heatmap results were obtained from the average of 5 independent replicates.

Binding free energies (BFE) were calculated for all the simulated systems using the molecular mechanics—generalized born surface area (MM-GBSA) method to estimate the binding affinity between the receptor and ligand. The total BFE (ΔG_bind_) was computed as ΔG_bind_ = E_Complex_ − E_Ligand_ − E_Receptor_. PRD analysis was performed to identify “hotspot” residues, revealing the contribution of individual amino acids to the overall ΔG_bind_. Snapshots were sampled from every fourth frame (7500 total frames), resulting in 1875 frames from the 3 μs simulation. The Prime module in Maestro was used (57, 58), employing the OPLS4 force field and the variable-dielectric generalized Born (VSGB) 2.1 solvation model (59).

To further characterize ligand dissociation and quantitatively describe the underlying free energy landscape, enhanced sampling MD simulations were performed. In this context, the potential of mean force (PMF) approach was employed, which is used to estimate free energy profiles along a defined reaction coordinate (RC) (60, 61). PMF is related to the Gibbs free energy as ΔG(x) = −k_BTlnP(x), where P(x) represents the probability density along the RC x, kB is the Boltzmann constant, and T is the absolute temperature. This framework was applied to evaluate the relative BFE profiles of A8535 and PT-91 with 3 fish SREB1 homologs. The RC was defined as the center-of-mass distance between the ligand and the receptor along the membrane (z-axis), corresponding to the ligand dissociation pathway. Additional information is provided in Supplemental File 1 (50).

### *In vitro* ovary treatments with PNX-20 and agonists

Wild-type adult zebrafish, mummichog, and pufferfish were maintained at the University of Florida (UF) Tropical Aquaculture Laboratory (TAL) (Ruskin, FL, USA) in the period 2024-2025. Briefly, zebrafish (0.73 ± 0.16 g, 39.71 ± 2.31 mm total length) were collected from freshwater earthen ponds on site at the TAL. Mummichog (6.20 ± 2.90 g, 80.2 ± 11.59 mm) were caught from the South Carolina Department of Natural Resources (Bluffton, SC, USA) and the Environmental Protection Agency: Atlantic Coastal Environmental Sciences Division (Narragansett, RI, USA). To verify mummichog used in this study was *F. heteroclitus*, fin clips (n = 2) were collected, shipped to University of Maine at Farmington (UMF) laboratories, and used in DNA extractions (Qiagen DNeasy Tissue Kit, Qiagen, Germany) with DNA sequencing of 2 mitochondrial CO1 regions (*mt-co1*) following standard procedures (62). Both individuals exhibited 2 *mt-co1* fragments with greatest similarity to *F. heteroclitus* (data not shown). Pufferfish (7.01 ± 2.13 g, 61.64 ± 5.05 mm) were imported through a nearby wholesaler (Segrest Farms, Inc., Gibsonton, FL, USA). All species were housed within an enclosed temperature-controlled greenhouse, and all studies were conducted according to UF Institutional Animal Care and Use Committee guidelines (IACUC #: IACUC202300000749). Zebrafish were held in a flow-through concrete vat supplied with treated well water (1060 L, temperature: 24.44 ± 3.33 °C, dissolved oxygen (DO): 7.11 ± 0.35 mg/L). Mummichog and pufferfish were held in a brackish water recirculating system (3513 L, salinity: 15-20 mg/L, temperature: 27.22 ± 0.56 °C, DO: 7.54 ± 0.51 mg/L).

To perform *in vitro* experiments, zebrafish (n = 17), mummichog (n = 20), and pufferfish (n = 14) were euthanized using 300 mg/L MS-222 (Syncainei^®^, tricaine methanesulfonate, Syndel, Ferndale, WA, USA) buffered 1:1 with sodium bicarbonate followed by pithing to ensure humane euthanasia. Paired ovaries were removed and partitioned into 5 approximately equal sections. The first ovary section was placed on a Sedgewick rafter cell and photographed with a Jenoptik ProgRes^®^ C5 microscope camera to characterize ovarian stages. Ovaries with the earliest developmental stages were preferentially selected, since previous research identified greatest SREB expression in early growth (39). Overall, zebrafish and mummichog ovaries exhibited minor populations of vitellogenic oocytes, but the majority of follicles in all 3 species were at primary and early secondary growth stages. Ovarian images from all individuals were assessed manually to ensure that none or minimal inclusions of early stage vitellogenic follicles were present, and all samples from each species were primarily at early developmental stages. The remaining 4 ovary sections were added to individual wells within a 24-well plate (Costar^®^ Cat#3524) with culture media (46). Each well contained 990 μL 90% Leibovitz's L-15 medium (Sigma Cat# L1518), 1% penicillin-streptomycin (Gibco™ Cat# 15070-063), 0.5% bovine serum albumin (Sigma Cat# 81053N), and 1 of the 4 treatments: (1) 100 nM PNX-20, (2) 5 μM A8535, (3) 5 μM PT-91, or (4) a negative dimethyl sulfoxide (DMSO) control. The 100 nM PNX-20 treatment was chosen based on previous zebrafish research (46). Species-specific and non-amidated (46) zebrafish, mummichog, and pufferfish PNX-20 were purchased from GenScript (≥98% purity, Piscataway, NJ, USA) with sequences of AGVNQADIQPVGVKVWSDPY, TGINQADVQPAGLKIWSDPF, and DGINQADVQPVGMKIWSDPF, respectively. Agonists A8535 and PT-91 were synthesized by the Pillaiyar laboratory (University of Tübingen, Germany) and confirmed to be of at least 95% purity by high-performance liquid chromatography and ^1^H and ^13^C nuclear magnetic resonance and high-resolution mass spectrometry. The 5 μM agonist treatment were chosen to avoid 10 μM solubility limits (33). To achieve final concentrations, PT-91 (4.5 mg) and A8535 (4.21 mg), were each dissolved in 1 mL of DMSO, generating 10 mM stock solutions, which were then diluted to 500 μM. PNX-20 (0.4 mg) was dissolved in 373.2 μL DMSO to achieve a 500 μM stock, which was then diluted to 10 μM. Agonist, PNX-20, and DMSO final stocks were added to each well containing culture media in 10 μL volumes.

Ovary fragments were incubated at 28 °C for 6 hours. Ovaries were then removed, preserved in RNAlater™ (Invitrogen Cat# AM7020), and snap frozen for RNA analyses. The remaining media was extracted using a glass pipette and placed in a glass vial for later steroidogenic analysis. To mitigate temporal variation, each *in vitro* experiment in all 3 species was equally represented at each time point over a 1 week period. All samples were stored at −80 °C.

### RNA extractions and cDNA synthesis

Total RNA was extracted using TRIzol™ (Invitrogen, Carlsbad, CA, USA; CAT#15596018) and standard phenol/chloroform procedures. To remove genomic DNA contamination, the TURBO DNA-free™ Kit (Invitrogen, catalog #AM1907) was used according to the manufacturer's protocol with minor modifications (2 μL of 10 × Turbo DNase™ Buffer/sample and 1.5 μL of Turbo DNase™ enzyme). Initial RNA quality was confirmed using the Qubit™ 4 Fluorometer (Invitrogen™, catalog #Q33238) and the Qubit™ RNA IQ Assay Kits (Invitrogen™, catalog #Q33222).

A total of 32 RNA samples per species were separated into 2 aliquots and shipped to (1) Novogene Corporation (Sacramento, CA, USA) for RNA sequencing (RNA-seq) and (2) UMF laboratories for quantitative PCR (qPCR) confirmation of RNA-seq patterns. At UMF, RNA quantity was assessed using a NanoDrop spectrophotometer and approximately 2.5 μg total RNA/sample was used for cDNA synthesis. Total RNA was DNase-treated using ezDNase with a 5-minute 37 °C incubation step followed by cDNA synthesis using the Superscript IV VILO kit (Invitrogen) and standard protocols. Stock cDNAs were stored at −20 °C.

### RNA sequencing

For each species, 16 samples (n = 4/treatment) with high RNA integrity numbers (RIN) were selected. Average RINs across all samples for zebrafish, mummichog, and pufferfish were 8.3 ± 0.16, 5.6 ± 0.28, and 8.0 ± 0.23, respectively. Mummichog RIN values were lower due to high proportions of small RNAs. To confirm this, fresh ovarian samples (n = 5) were used in RNA extractions and quality assessed on an Agilent 4150 TapeStation (Santa Clara, CA, USA), with similar results (6.5 ± 0.2). Poly A enrichment was conducted at Novogene (Sacramento, CA, USA), and individually paired libraries were pooled by equimolar concentrations and sequenced using NovaSeq X Plus Series with 150 bp paired-end reads (Illumina Inc., CA, USA). Raw reads of FASTQ format were initially processed through fastp. Clean data/reads were obtained from raw data by filtering out reads with adapter sequences, poly-N sequences, and reads of low quality. Q20, Q30 and GC content of the clean data were determined and provided in Tables S2-S4 (50). Only high-quality clean data were used in downstream analyses.

Reference genomes were downloaded from NCBI for each species (zebrafish GRCz11; mummichog MU_UCD_Fhet_4.1; pufferfish Proteome ID UP000007303). HISAT2 software was used to map paired-end clean reads to the reference genome. Feature counts were used to count read numbers mapped to each gene, including both known and novel genes. Reads per kilobase of transcript, per million mapped reads of each gene was calculated based on the gene length and read count mapped. Ovary transcriptomes from zebrafish (GSE291115), mummichog (GSE313040), and pufferfish (GSE299271) were submitted to the NCBI Gene Expression Omnibus database. DESeq2 was used for differential expression analysis within species between the negative control and each treatment. Differentially expressed genes (DEGs) with log_2_ fold changes and *P*-values (*P*_adj._ and raw *P*-value) are provided in Supplemental File 2 (63). To identify if treatment exposures induced significantly more genes to be up- or downregulated in each species, total DEG lists (*P* < .05) were assessed using χ^2^ analyses. Observed DEGs were compared to expected numbers assuming equally significant up- and downregulation in each treatment.

### Pathway analysis and meta-analyses

Pathway analyses were conducted using iPathwayGuide (Advaita Bioinformatics, MI, USA). Briefly, DEGs were mapped to human homologs and filtered using a threshold of *P* < .05 and absolute log_2_ fold changes of at least 0.6. Data were analyzed using (1) pathways obtained from the Kyoto Encyclopedia of Genes and Genomes (KEGG) database (64), (2) gene ontologies (GO) from the Gene Ontology Consortium database (65), (3) miRNAs from the miRBase (MIRBASE version 22.1, October 2018) and TARGETSCAN database (versions 8.0 for mouse and human) (66), (4) network of regulatory relations from BioGRID: Biological General Repository for Interaction Datasets v4.4.233, April 25, 2024 (67), (5) chemicals/drugs/toxicants from the Comparative Toxicogenomics Database October 2023 (68), and (6) diseases from the KEGG database (Release 110.0+/05-01, May 24) (64). Approximately 12 300, 10 600, and 12 000 genes with measured expression from zebrafish, mummichog, and pufferfish, respectively, were assessed.

Significant changes were compared to the negative control and assessed by both false discovery rate (FDR) and raw *P*-value (*P* < .05). Since several comparisons exhibited extensive gene ontology lists, GO molecular functions (MFs) and biological processes (BPs) were listed only by FDR for individual species (Supplemental File 3) (69). To better visualize patterns across BPs, the Reduce and Visualize Gene Ontology (REVIGO) program was used to cluster entries based on semantic similarity (70). REVIGO uses a clustering algorithm to reduce functional redundancy and simplify BPs into 2-dimensional plots (70). In total, only 3 treatments (zebrafish A8535, zebrafish PT-91, and pufferfish PNX-20) exhibited >2 BPs significant by FDR and could be visualized (see Supplemental File 3) (69). Clusters with similar terms were highlighted using a manually assigned label based on collective functions.

Meta-analyses of DEGs, pathways, and BPs were conducted by 2 approaches. First, pathways and all BPs (*P* < .05) significantly altered by multiple treatments within each species were identified using iPathwayGuide. Upset plots were generated to visualize pathways across treatments (71). Pathways and BPs found across multiple treatments are listed in Supplemental File 3 (69). BPs generated from these comparisons were also assessed in REVIGO. Second, DEGs and pathways (*P* < .05) significantly altered by one treatment and found across multiple species were identified manually, and pathways were confirmed using iPathwayGuide. Since gene annotations from genomic databases differed across species, similar (eg, *abca4* vs *abca4b*) or identical gene abbreviations (eg, *cxc18a* vs *cxcl18a*) were counted as the same gene for each comparison.

### Quantitative PCR

To confirm RNA-seq patterns, selected DEGs were used in relative quantitative PCR (qPCR) assays. All species were used with FAST SYBR™ Green Master Mix following standard protocols (Applied Biosystems, Foster City, CA, USA). Zebrafish and pufferfish assays were run in a StepOnePlus Real Time PCR system, while mummichog assays were run in a QuantStudio 3 Real-Time PCR system (Applied Biosystems). For zebrafish, target genes included *caprin1a*, *rpl36a*, *sec11a*, *steap4*, *tmsb4x*, and *wu:fj16a03*, and primers were designed from available sequences in NCBI GenBank (NM_001003484, NM_001202510.1, NM_001002521.2, NM_001017734.2, NM_001130697.1, and NM_001308554.1, respectively). For mummichog, target genes included C18orf32, LOC105926222, LOC110369011, LOC118566921, *nd1*, *rps2*, and *slc45a2*, and primers were designed from GenBank entries XM_036140262.1, XR_004932134.1, XR_004927763.1, XR_004933358.1, NC_012312.1, XM_036148098.1, and XM_012879750.3, respectively. For pufferfish, target genes included *celsr2*, *cryaa*, *gm2a*, *gpr179* (labeled as *gpr158* in DEGs), *sema3d*, *tuba4a*, and *xdh*, and primers were designed from gene sequences in the Ensembl database (TETRAODON 8.0). Three commonly used reference genes were also used (18S rRNA, *eef1a*, *gapdh*). Zebrafish and pufferfish reference gene primers were previously designed (37, 72). Mummichog 18S rRNA and *eef1a* were also previously designed (38), but *gapdh* was designed from XM_012870348.3. All primer sets were first verified for intended product amplification (62). All primer sequences are available in Tables S5-S7 (50).

All qPCR assays were performed using standard parameters with 50 ng/μL bovine serum albumin and standard cycling conditions with dissociation curve analysis (38, 39, 73). To verify 90-110% PCR efficiency, standard curves were generated. All assays exhibited single peak amplification, and standard qPCR negative controls exhibited no contamination. Results were analyzed using a relative quantitative approach (74) and normalized across all 3 reference genes that exhibited no significant differences across treatments in any species. All normalized results were expressed relative to the negative control (set to 1.0), and data were expressed as mean ± standard error. To meet assumptions of normality, qPCR data were log-transformed and analyzed using 1-way ANOVAs in SYSTAT12 (Systat Software Inc., San Jose, CA, USA). To identify significant regressions (*P* < .05) between qPCR and RNA-seq data, log-transformed relative expression values and normalized RNA-seq counts were also compared using least-squares regression analyses.

### Steroid quantifications

Frozen media samples were shipped to the UF Center for Environmental and Human Toxicology Analytical Toxicology Core Laboratory (ATCL, Gainesville, FL, USA). Steroids were quantified via a liquid chromatography tandem mass spectrometry approach previously validated for low volumes (75). The SCIEX 7500+ system low-mass spectrophotometer (Framingham, MA, USA) was used with the ExionLC AE liquid chromatography system (Framingham, MA, USA). Injection volumes were 7 μL. Five steroids were quantified ([17 estradiol [E2], 17OH-progesterone [17OHP], cortisol [F], testosterone [T], and 11-ketotestosterone [11-KT]). Steroid quantities from extracts (100 μL) were normalized to whole media volume, then normalized to ovary mass (g). Samples under the instrument's limit of detection (LOD) or below limit of quantitation were replaced with the lowest detection value. This was calculated using the instrument LOD based on ng/mL extract and was modified to an LOD value normalized to the whole media volume (LODmedia [ng/mL] = LODextract [ng/mL] [Vextract [μL]/Vmedia [μL]). Steroid quantifications included the same individuals as assessed in RNA-seq, and most experiments included an additional sample to increase sample sizes. In total, zebrafish and pufferfish treatments included n = 5/treatment/species, while mummichog control and A8535 treatments varied (n = 6 and 4, respectively). Normalized steroid quantities (ng/g tissue) were log-transformed and used in 1-way ANOVAs in SYSTAT12.

## Results

### *In silico* characterization of agonist interactions with fish SREB1s

As there are currently no experimentally resolved structures available for SREB1 in the 3 fishes, HMs were generated using prior work with the human receptor as a template with PT-91 (36). Superposition of the generated HMs with the human reference structure demonstrated high overall structural conservation, particularly within the transmembrane domains (Fig. 1). The structural similarities were quantified using RMSD against the reference human SREB1. The resulting RMSD values were highly favorable: 1.2171 Å for zebrafish, 1.4269 Å for mummichog, and 1.0388 Å for pufferfish, indicating that the currently built models are highly reliable and suitable for subsequent *in silico* ligand-binding studies.

Molecular docking was performed to predict the binding poses of A8535 against the reference PT-91. Docking of ligand A8535 was considered highly successful, as it occupied a binding pocket similar to that of PT-91 across all 3 fishes (Fig. S2, Table S8) (50). The primary selection criterion for the optimal docking pose was the ability of compound A8535 to replicate the binding mode and key molecular interactions observed with PT-91. As illustrated in the 2D interaction diagrams (Fig. S2) (50), both ligands were stabilized primarily through a network of robust hydrophobic interactions, π-π stacking, and specific hydrogen bonds, confirming that A8535 acts within the same orthosteric site as PT-91.

**Figure 2:**
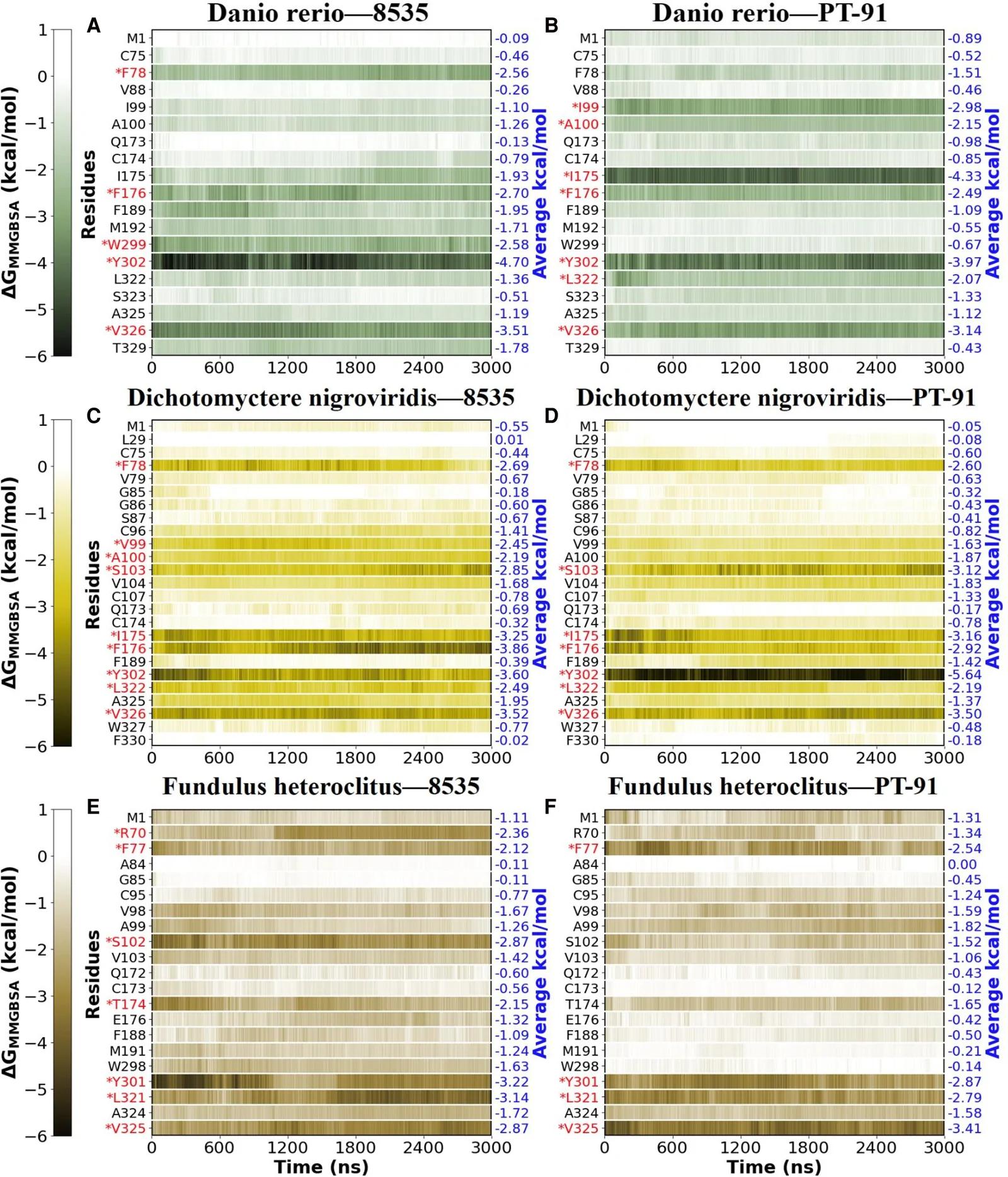
PRD (represented in kcal/mol) heatmaps displaying the energy contributions of binding pocket residues over time (3 μs). Here, all the heatmaps display the BFE contributions (ΔG in kcal/mol) calculated via MM-GBSA over the full 3 μs MD trajectories. Panels represent the following 12 complexes: *D. rerio* with (A) ligand 8535 and (B) PT-91; *D. nigroviridis* with (C) 8535 and (D) PT-91, (E) *F. heteroclitus* with 8535, and (F) PT-91, respectively. The x-axis represents simulation time, indicating the temporal stability of the residue interactions. Darker horizontal bands indicate residues that consistently provide strong favorable (negative) energy contributions to ligand binding. The right-hand y-axis (blue values) lists the time-averaged energy contribution for each residue. Residues marked with an asterisk (*) denote the most significant “hotspot” residues driving the ligand–receptor affinity.

**Figure 3:**
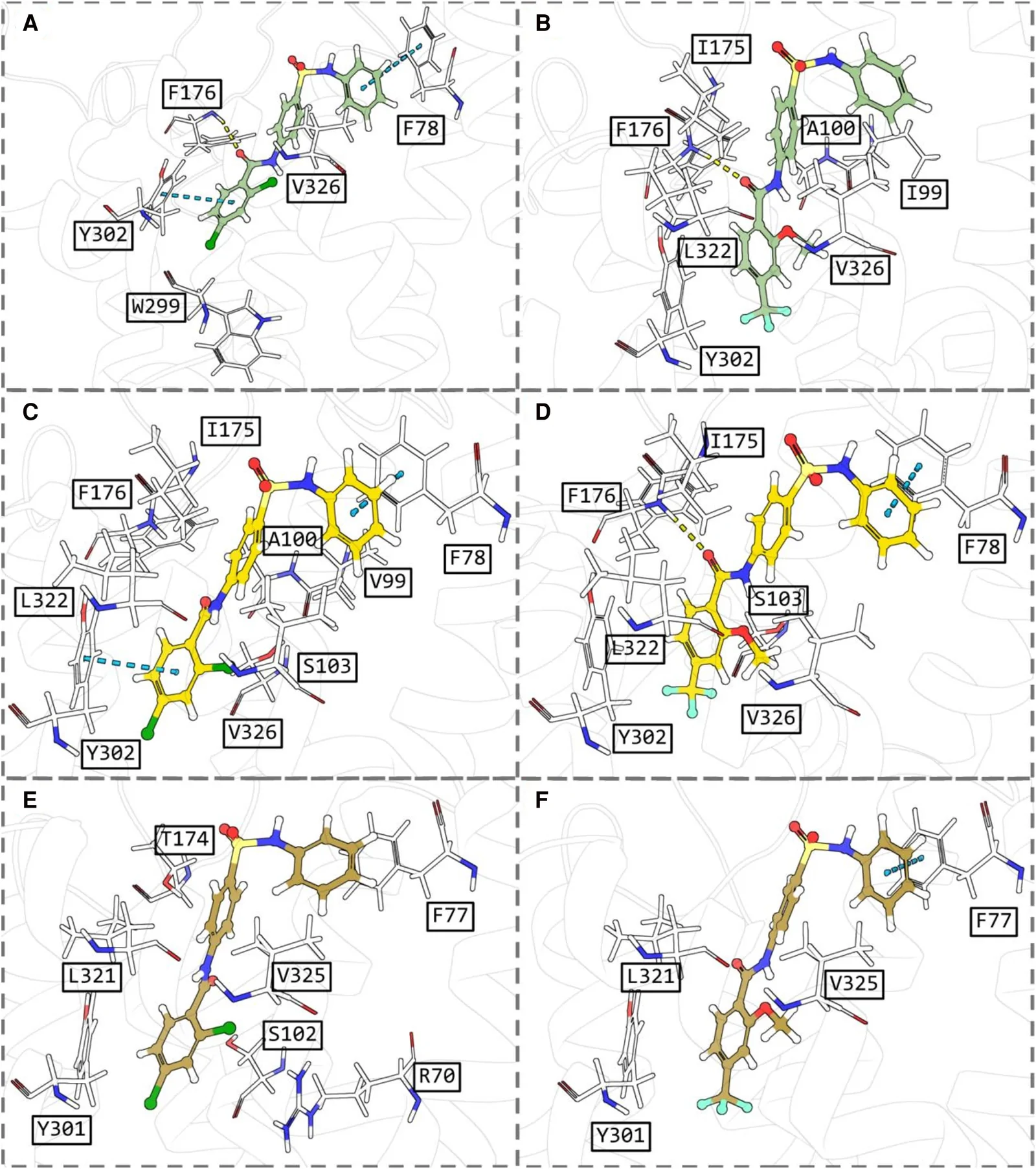
Three-dimensional visualizations of the orthosteric binding pocket highlighting hotspot residues. The structural positioning of the key energy-contributing residues was identified through the PRD analysis in Fig. 2. The panels correspond to the receptor–ligand systems: zebrafish (*D. rerio*) with ligand A8535 (A) and PT-91 (B); pufferfish (*D. nigroviridis*) with A8535 (C) and PT-91 (D); and mummichog (*F. heteroclitus*) with A8535 (E) and PT-91 (F). The ligands and interacting hotspot residues (labeled in boxes) are rendered in stick-and-ball representation. Yellow dashed lines represent hydrogen bonds and cyan dashed lines represent π-π interactions.

To identify the hotspot amino acids responsible for ligand recognition, PRD analysis was conducted from the total BFE (ΔG_bind_) profiles. The interaction frequency analyses (Fig. S3, Table S9) (50) and PRD heatmaps (Fig. 2, Table S10) (50) highlighted several highly correlated residues that consistently provided significant energy contributions. Specifically, homologous tyrosine residues (Y302 in zebrafish/pufferfish and Y301 in mummichog), phenylalanine residues (F78/77), and valine residues (V326/325) showed high interaction frequencies and consistently high energy contributions (predominantly through hydrophobic and π-π packing), marking them as critical anchors for ligand recognition in SREB1s (Table S10) (50). Three-dimensional visualizations of SREB1 orthosteric binding pockets and identified hotspot residues are presented in Fig. 3.

**Figure 4:**
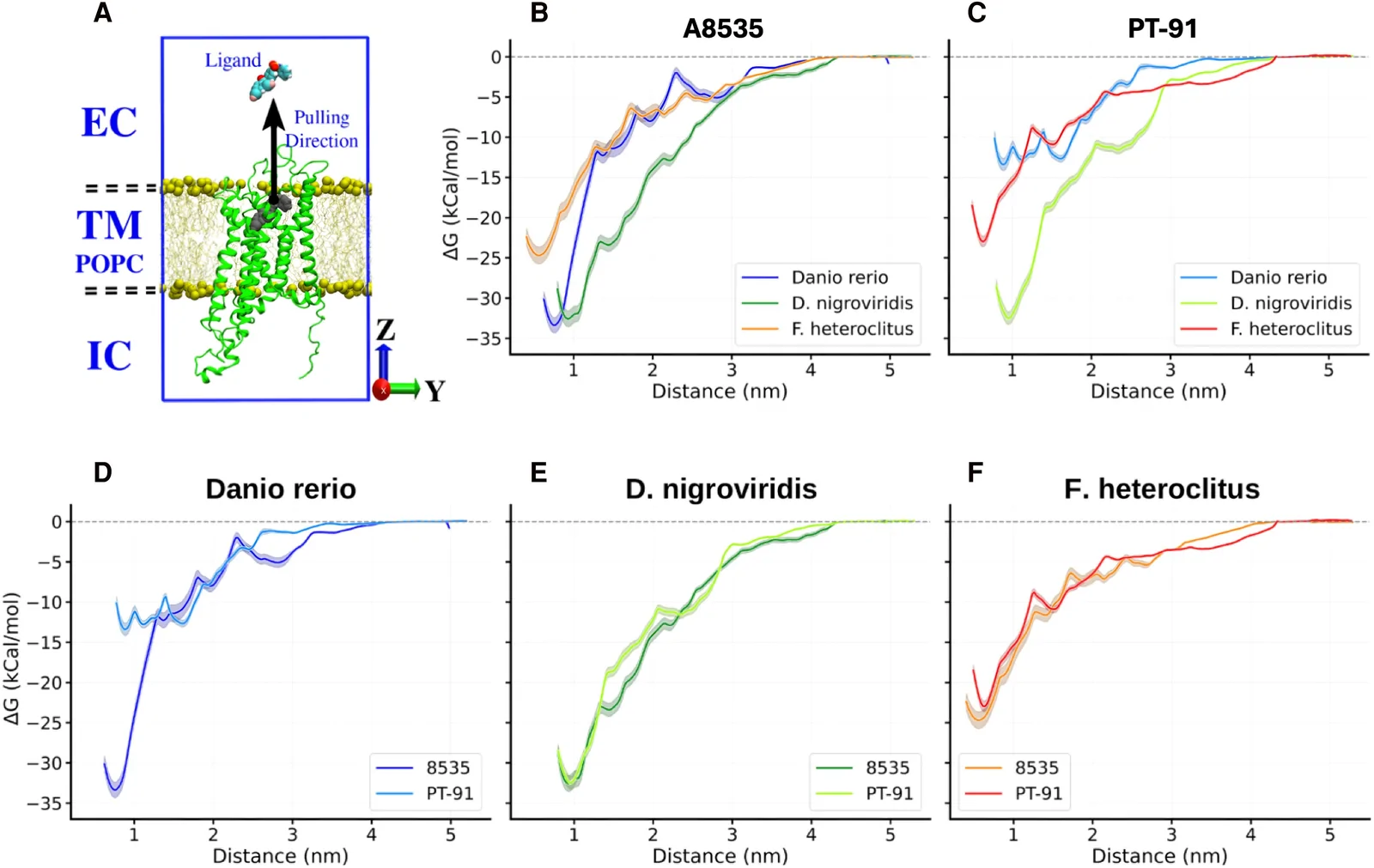
Schematic representation of the umbrella sampling setup (A). The receptor (green) is embedded in a POPC lipid bilayer (yellow), with the ligand being pulled from the orthosteric binding pocket along the membrane normal (z-axis) toward the ECL space. TM and IC denote transmembrane and ICL regions, respectively. Ligand-wise PMF comparison for ligands A8535 (B) and PT-91 (C). Receptor-wise comparison of both ligands in zebrafish (*D. rerio*, D), pufferfish (*D. nigroviridis*, E), and mummichog (*F. heteroclitus*, F). Shaded areas represent statistical error estimates. Energies are represented in kcal/mol.

To extend these findings beyond static interaction energies by the PRD analysis, PMF calculations were performed to capture the full free energy landscape of ligand binding and dissociation. The resulting free energy profiles provide insights into both the thermodynamic stability of the bound state and the energetic barriers associated with ligand dissociation. Initially, the PMF graphs were investigated along the ligand-wise perspective (Fig. 4A-4C, Fig. S4) (50), which highlighted a distinct difference in BFE profiles between the 2 ligands across the 3 receptor systems. For ligand A8535, all 3 receptors exhibited deep global minima located at short ligand–receptor distances (∼0.7-1.2 nm). Among them, the deepest minimum was observed for zebrafish, reaching −33.38 ± 0.96 kcal/mol, followed closely by pufferfish (−32.57 ± 0.99 kcal/mol). In contrast, mummichog showed a comparatively shallower minimum (−24.70 ± 1.05 kcal/mol). For PT-91, pufferfish exhibited the most favorable binding (−32.51 ± 0.79 kcal/mol), with a global minimum comparable to that of A8535. However, in zebrafish, the BFE was significantly reduced compared to A8535, with a shallower minimum (−13.40 ± 0.77 kcal/mol). Mummichog displayed an intermediate binding strength (−22.97 ± 0.65 kcal/mol).

Additionally, the PMF curves were also compared along the receptor-wise perspectives (Fig. 4D-4F) to compare ligand-specific differences within each species. In zebrafish, a marked difference between the 2 ligands was observed. A8535 exhibited a substantially deeper free energy minimum compared to PT-91. In pufferfish, both agonists displayed similarly deep minima. In mummichog, the 2 agonists exhibited nearly identical PMF profiles, with minima around −22 to −24 kcal/mol. These PMF profiles indicate that ligand selectivity is highly species-dependent, with zebrafish demonstrating a distinct pattern for PT-91, whereas mummichog and pufferfish receptors interact with both agonists nonselectively but at markedly different overall binding affinities.

### Transcriptomic changes in zebrafish ovaries

**Figure 5:**
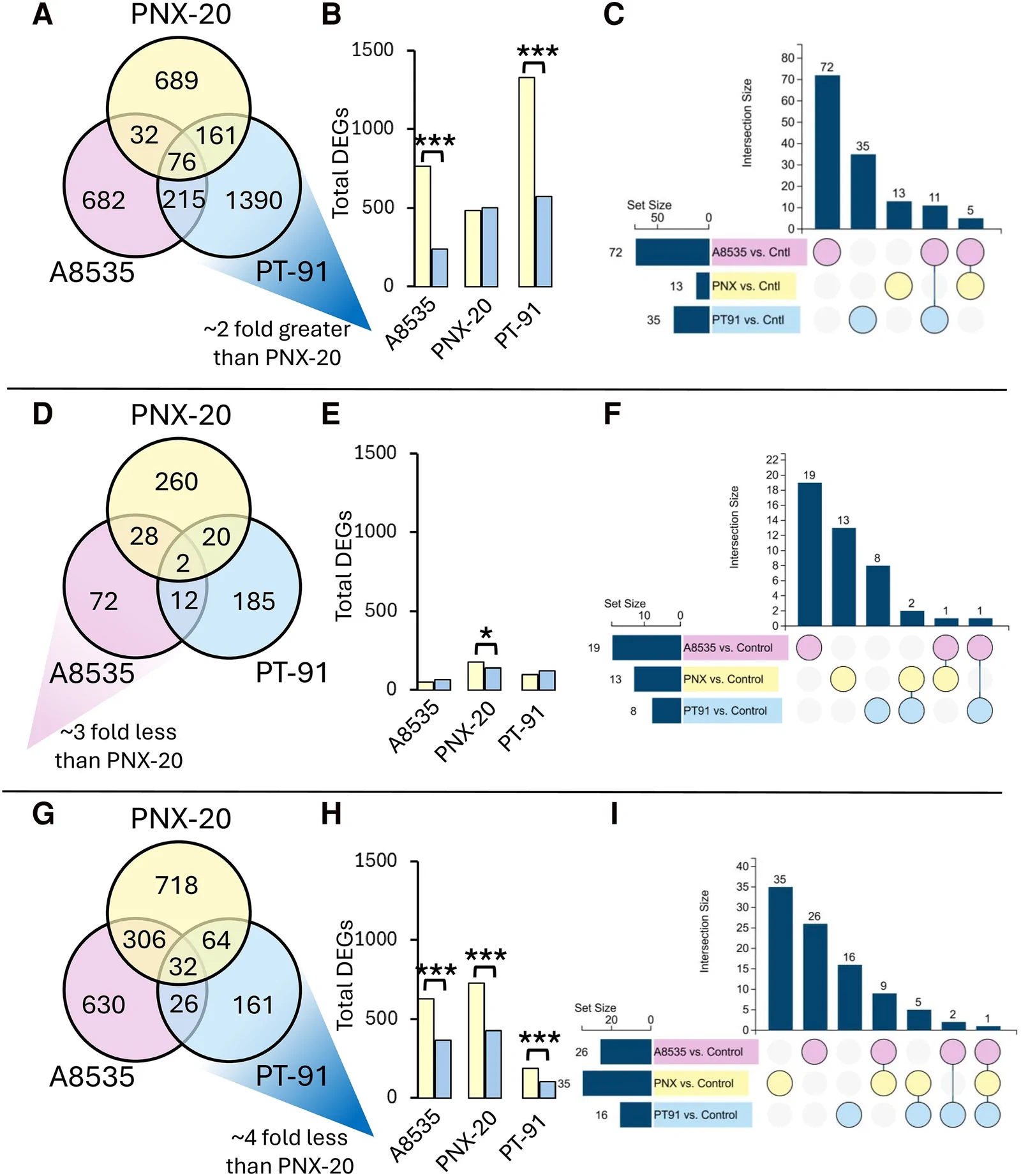
Venn diagram of DEGs identified in RNA-seq (left), total DEGs up- or downregulated (center), and upset plots of significantly altered gene pathways in each treatment (right) in zebrafish (A-C), mummichog (D-F), and pufferfish (G-I). For Venn diagrams (A, D, and G), numbers refer to the total DEGs (*P* < .05) identified following phoenixin (PNX-20, yellow), A8535 (pink), and PT-91 (blue) ovary treatments in each species. Common DEGs among treatments are labeled in each intersecting region, and colored triangles (blue or pink) underneath a treatment highlight a notable fold increase or decrease in relation to an endogenous hormone (PNX-20) response. For graphs of total DEGs up- (yellow) or downregulated (blue) (B, E, and H), significance was assessed at *P* < .05 (*) and <.001 (***) levels. For upset plots (C, F, and I), intersection and set sizes refer to the total number of pathways significantly altered in each treatment (yellow = PNX-20, pink = A8535, and blue = PT-91; *P* < .05). Number of common pathways shared across multiple treatments are indicated by colored circles with connecting lines.

Zebrafish ovaries exposed to PNX-20, A8535, and PT-91 exhibited significant changes (*P* < .05) in 958, 1004, and 1842 genes, respectively (Fig. 5A). Overall, PT-91 induced the greatest response and exhibited approximately twice as many changes as those induced by the endogenous hormone (PNX-20). The agonists exhibited the most overlap in gene changes (215), while the least similarity was evident between A8535 and PNX-20 (Fig. 5A). PNX-20 responses were also divergent from the agonists in terms of up- and downregulated gene patterns, where both A8535 and PT-91 induced significantly more upregulation than expected (χ^2^ = 277.67 and 290.89; *P* < .001 and <.0001, respectively) while PNX-20 was balanced (*P* > .05, Fig. 5B). When DEGs were sorted into pathways (*P* < .05), the most diversity in functional responses was evident in the A8535 treatment, with a total of 72 unique pathway changes (Fig. 5C). In contrast, PT-91 exposure altered approximately half as many (35 unique pathways), and PNX-20 exhibited the least diversity.

Reduced PNX-20 effects compared to agonists were also evident in specific pathways, MFs, and BPs. PNX-20 did not induce any highly significant changes in pathways or GO terms at the FDR level (Table 1). However, some weaker pathway changes (*P* < .05) were evident in the PNX-20 treatment, including DNA replication (*P* = .0148), renin secretion (*P* = .0166), and glutaminergic synapse (*P* = .0230).

**Table 1:** Selected zebrafish pathways and gene ontology (GO) processes significantly altered in ovaries treated *in vitro* with phoenixin (PNX-20), agonist A8535, or agonist PT-91

	PNX-20	A8535	PT-91
Pathways—FDR	None	Focal adhesion (0.0161)	**Ribosome (<0.0001)**
		ECM–receptor interaction (0.0216)	**Oxidative phosphorylation (<0.0001)**
		TGF-beta signaling pathway (0.0499)	RNA polymerase (0.0024)
Pathways—*P* < .05	DNA replication (0.0148)	Cell adhesion molecules (0.0078)	Cardiac muscle contraction (0.0031)
	Renin secretion (0.0166)	Ovarian steroidogenesis (0.0111)	Motor proteins (0.0094)
	Glutaminergic synapse (0.0230)	Insulin secretion (0.0497)	Metabolism of xenobiotics by cytochrome P450 (0.0299)
MF—FDR	None	**ECM structural constituent (<0.0001)**	**Structural constituent of ribosome (<0.0001)**
		Integrin binding (0.0064)	**Oxidoreduction-driven active** **Transmembrane activity (<0.0001)**
		Cell adhesion molecule binding (0.0165)	**Electron transfer activity (<0.0001)**
BP—FDR	None	**Anatomical structure formation involved in morphogenesis (<0.0001)**	**Oxidative phosphorylation (<0.0001)**
		**Blood vessel morphogenesis (<0.0001)**	**Aerobic respiration (<0.0001)**
		**Regulation of cell migration (<0.0001)**	**Cytoplasmic translation (<0.0001)**

Pathways are separated into entries significant by FDR or *P* < .05. Only GO MF and BP terms are shown that meet significance by FDR. Up to 3 entries were chosen for each category based on (1) approximately 10% or higher representation in DEGs relative to total gene count in a network and (2) exclusion of immune-related processes. All significantly altered pathways and GO terms can be found in Supplemental File 3 (69). Bolded terms refer to highly significant entries (*P* < .0001).

**Figure 6:**
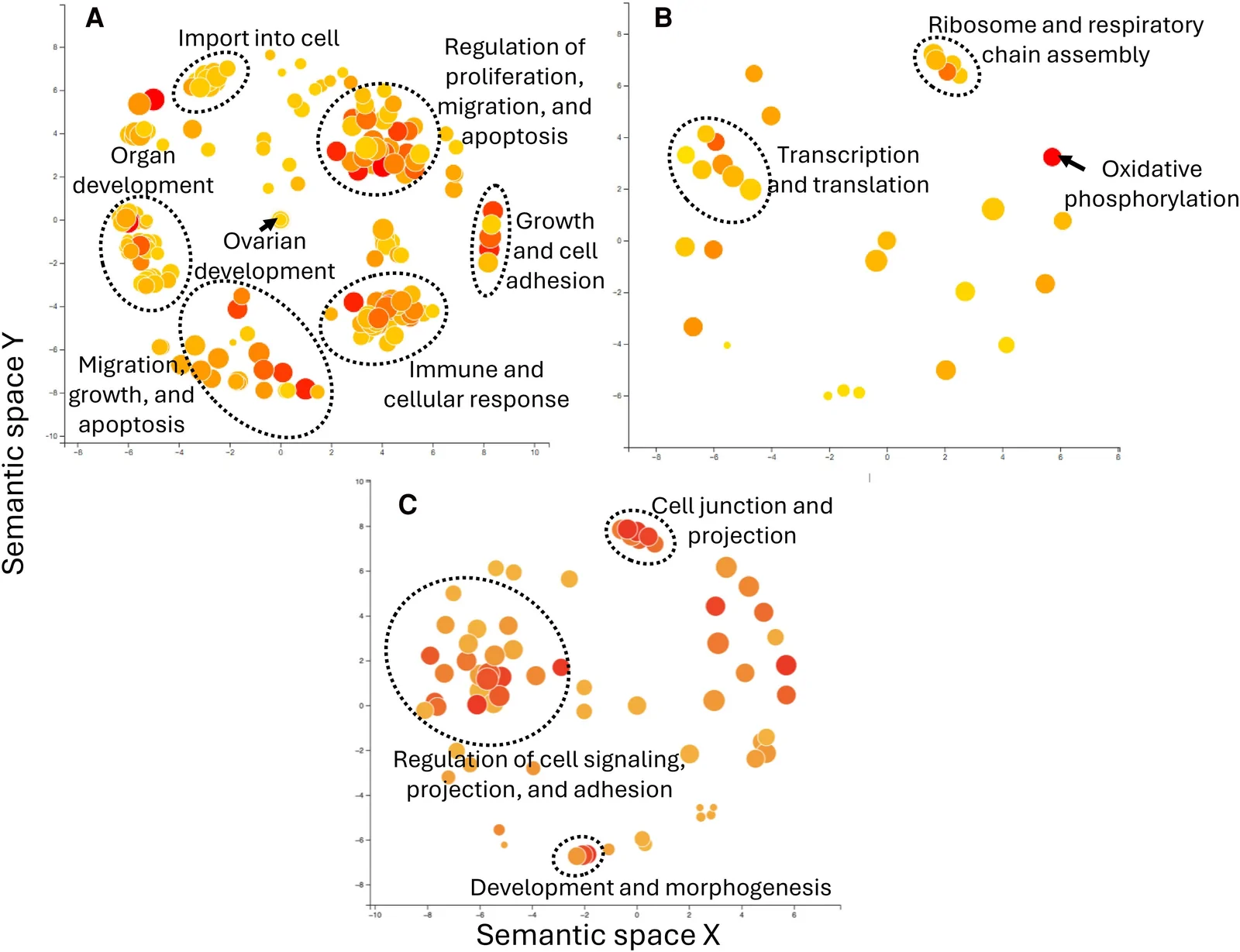
Highly significant GO BP terms clustered into functional groupings using semantic similarity in REVIGO. Dotted black circles refer to manual assignment of term clusters with similar functions. Zebrafish A8535 (A), zebrafish PT-91 (B), and pufferfish PNX-20 (C) ovary treatments are shown. Other treatments and species are not shown since they exhibited less than 3 highly significant BPs by FDR. Darker and larger bubbles refer to terms with greater significance and more generality in the GO database, respectively. Selected BPs are also highlighted with arrows. Complete lists for each species and treatment are available in species-specific BP tabs in Supplemental File 3 (69).

In contrast, A8535 exposure induced 15 and 57 pathway changes at the FDR and *P* < .05 levels, respectively, which included highly significant changes in focal adhesion and ECL matrix (ECM)–receptor interactions (*P* = .0161 and .0216, respectively) (see Table 1 for selected pathways). Weaker pathway changes in A8535 included ovarian steroidogenesis and insulin secretion (*P* = .0111 and .0497, respectively). With respect to ovarian steroidogenesis, mapping of genes involved in this pathway revealed weak upregulation of several with known reproductive roles in follicular cells, including the luteinizing hormone receptor (*lhcgr*), aromatase (*cyp19a1a*), and 3-beta hydroxysteroid dehydrogenase (*hsd3b*) (see Fig. S5) (50). Finally, A8535 treatment induced highly significant changes in 8 and 407 MFs and BPs, respectively. Some of the most significant changes included ECL matrix structural constituent (MF) and anatomical structure formation involved in morphogenesis (BP) (*P* < .0001, Table 1). When BPs were grouped based on semantic similarity, A8535 treatment exhibited clusters related to cell import, regulation of cell migration, growth, and apoptosis, and organ development (Fig. 6A).

**Figure 7:**
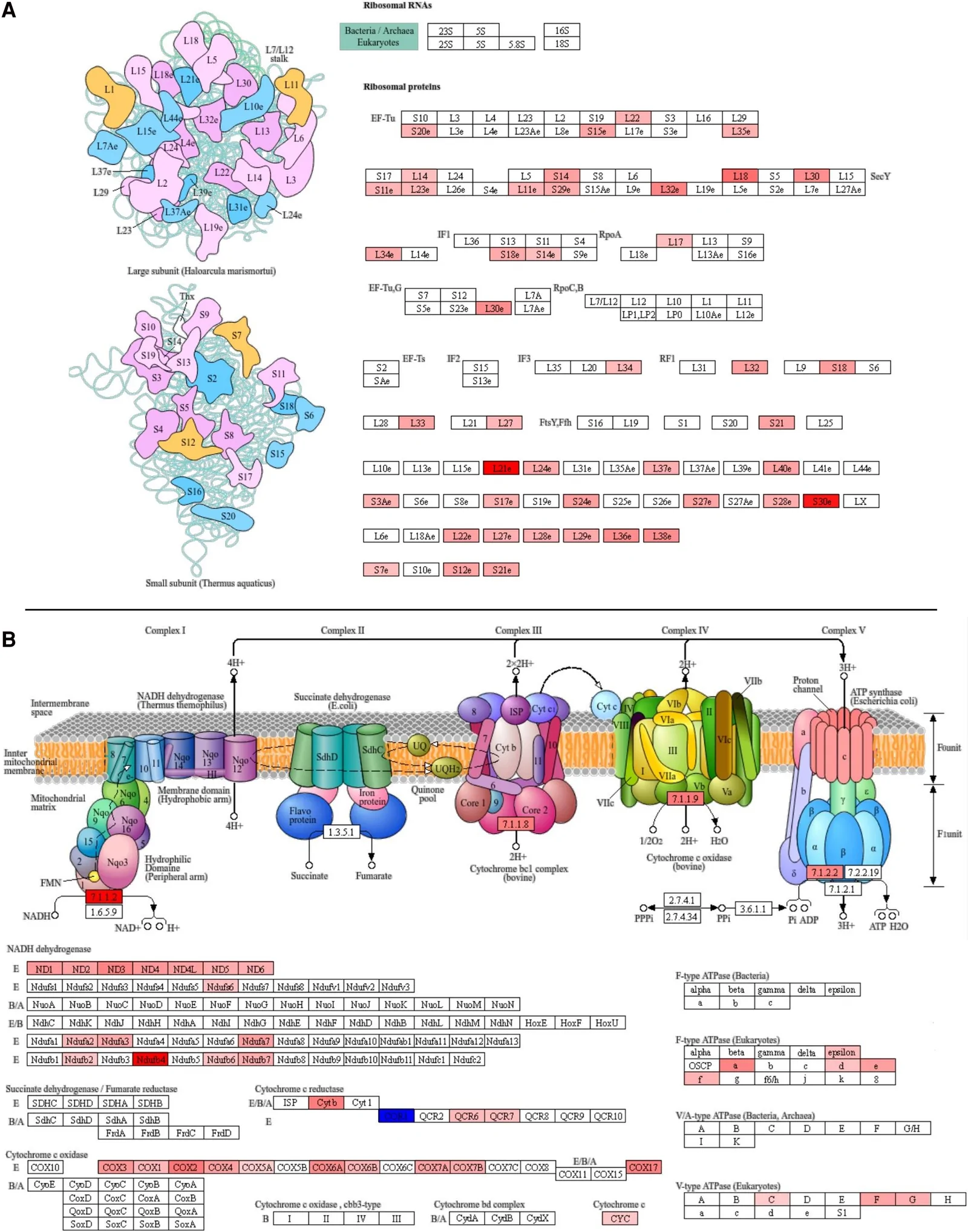
Pathway diagrams for ribosome (A) and oxidative phosphorylation (B) identified in the zebrafish ovary following PT-91 treatment (*P* < .0001). Red and blue blocks refer to genes up- or downregulated in each pathway, respectively.

PT-91 treatment induced 15 and 20 pathway changes in zebrafish that were significantly altered at the FDR and *P* < .05 levels, respectively (see Supplemental File 3) (69). Many focused on energy metabolism and protein synthesis. These included highly significant changes in ribosome, oxidative phosphorylation, and RNA polymerase (*P* < .0001, <.0001, and .0024, respectively, Table 1). Mapping of these pathways demonstrated upregulation of 43 total genes related to ribosomal proteins and 42 genes related to oxidative phosphorylation, which included all NADH dehydrogenases (*nd*), 10 cytochrome c oxidase genes, and others associated with complex I, III, IV, and V (Fig. 7). Additionally, PT-91 treatment also exhibited weaker pathway changes, including cardiac muscle contraction and motor proteins (*P* < .0031 and .0094, respectively). Highly significant MFs and BPs (28 and 66, respectively) also exhibited similar patterns to pathways, with greatest changes related to the ribosome and electron transfer activity (MFs, *P* < .0001), or aerobic respiration and cytoplasmic translation (BPs, *P* < .0001). When clustered, these BPs exhibited clear groupings related to transcription/translation, ribosome, and respiratory chain assembly (Fig. 6B).

### Transcriptomic changes in mummichog ovaries

Mummichog ovaries exhibited the least overall changes. When exposed to PNX-20, A8535, and PT-91 treatments, changes were evident in 310, 114, and 219 DEGs, respectively (Fig. 5D). While PNX-20 and PT-91 were fairly similar in terms of DEG number, A8535 exposure induced very few changes and approximately threefold less DEGs compared to the endogenous response (Fig. 5D). In contrast to zebrafish, PNX-20 was the only treatment with significantly more upregulated genes than downregulated in mummichog (**χ**^2^ = 4.18, *P* < .05) while A8535 and PT-91 exhibited largely equal proportions (Fig. 5E). Pathways altered in mummichog were also relatively low, with 19, 13, and 8 significant pathway changes in treatments A8535, PNX-20, and PT-91, respectively (Fig. 5F).

PNX-20 induced few pathway changes in the mummichog ovary, with no pathways identified by FDR (see Supplemental File 3) (69). Weaker changes in pathways included phototransduction (*P* = .0017), pancreatic secretion (*P* = .0197), and thyroid hormone signaling (*P* = .0332) (Table 2). The very few highly significant MFs and BPs identified were involved in cardiac and vascular regulation (Table 2).

**Table 2:** Selected mummichog pathways and GO processes significantly altered in ovaries treated *in vitro* with phoenixin (PNX-20), agonist A8535, or agonist PT-91

	PNX-20	A8535	PT-91
Pathways—FDR	None	Long-term potentiation (0.0385)	None
		cAMP signaling pathway (0.0448)	
Pathways—*P* < .05	Phototransduction (0.0017)	Pancreatic secretion (0.0067)	Phototransduction (0.0006)
	Pancreatic secretion (0.0197)	Glutamingeric synapse (0.0134)	Neuroactive ligand-receptor interaction (0.0033)
	Thyroid hormone signaling pathway (0.0332)	VEGF signaling pathway (0.0283)	cAMP signaling pathway (0.0394)
MF—FDR	VEGF receptor activity (0.0434)	Glutamate-gated receptor activity (0.0485)	Potassium channel activity (0.0085)
			Sodium channel activity (0.0134)
		Glutamate receptor activity (0.0485)	Potassium ion transmembrane transporter activity (0.0134)
		Calcium-dependent phospholipid binding (0.0485)	
BP—FDR	Positive regulation of heart contraction (0.0406)	None	None
	Positive regulation of blood circulation (0.0406)		

Pathways are separated into entries significant by FDR or *P* < .05. Only GO MF and BP termed are shown that meet significance by FDR. Up to 3 entries were chosen for each category based on (1) approximately 10% or higher representation in DEGs relative to total gene count in a network when able and (2) exclusion of immune-related processes. All significantly altered pathways and GO terms can be found in Supplemental File 3 (69).

In the A8535 treatment, 2 pathways were highly significant, including long-term potentiation (*P* = .0385) and cAMP signaling (*P* = .0448). At the *P* < .05 level, 17 pathways were weakly altered and included pancreatic secretion (*P* = .0067) and vascular endothelial growth factor (VEGF) signaling (*P* = .0283, Table 2). A total of 25 MFs were highly significant and included glutamate receptor activity (*P* = .0485). In contrast, no BPs were highly significant (Table 2).

No pathways were significantly altered at the FDR level following PT-91 treatment (Supplemental File 3) (69). Weaker changes included neuroactive ligand–receptor interaction and the cAMP signaling pathways (*P* = .0033 and .0394, Table 2). Significant MFs by FDR in PT-91 (3 total) contributed to sodium and potassium transport. There were no highly significant BPs following treatment with PT-91.

### Transcriptomic changes in pufferfish ovaries

Pufferfish exhibited intermediate changes, with PNX-20, A8535, and PT-91 treatments inducing 1120, 994, and 283 DEGs, respectively (Fig. 5G). PT-91 exhibited comparatively few gene expression changes, which was almost fourfold less than the endogenous response. There was also considerable overlap (306 DEGs) between PNX-20 and A8535 treatments, which was greater than other species. All 3 experimental treatments induced significantly greater proportions of upregulated genes in pufferfish (χ^2^ = 75.09, 69.06, and 23.18, respectively, with *P* < .001, Fig. 5H). The greatest pathway diversity was reflected in the PNX-20 treatment (35 pathways), while A8535 altered 26 unique pathways, and PT-91 exhibited 16 pathways (Fig. 5I).

PNX-20 treatment in pufferfish ovary did not induce highly significant changes in any pathway. However, weaker changes (*P* < .05) included type II diabetes (*P* = .0002); growth hormone synthesis, secretion, and action (*P* = .0070); and GnRH secretion (*P* = .0172) (Table 3). GnRH secretion was significantly altered in 2 treatments (PNX-20 and A8535), and pathway mapping demonstrated upregulation of several genes associated with potassium and calcium signaling, including *kir* and both T-type, and L-type calcium channels. Other genes associated with calcium signaling, including phospholipase C (*plc*), phosphatidylinositol 3-kinase (*pi3k*), and *cacna1g* (encoding Ca_v_3.1 calcium channel), were also involved (Fig. S6) (50). PNX-20 induced highly significant changes in MFs and BPs (8 and 114, respectively, Supplemental File 3) (69). Many of these were related to histone modifying processes, cell junction assembly, and development. In particular, cell morphogenesis was highly significant following PNX-20 treatment (*P* < .0001, Table 3). When all of these BPs were clustered by semantic similarity, clear groups were identified based on cell signaling, junctions, and projection along with morphogenesis and development (Fig. 6C).

**Table 3:** Selected pufferfish pathways and GO processes significantly altered in ovaries treated *in vitro* with phoenixin (PNX-20), agonist A8535, or agonist PT-91

	PNX-20	A8535	PT-91
Pathways—FDR	None	None	None
Pathways—*P* < .05	Type II diabetes mellitus (0.0002)	Growth hormone synthesis, secretion and action (0.0016)	Drug metabolism—other enzymes (0.0183)
	Growth hormone synthesis, secretion and action (0.0070)		
		Cortisol synthesis and secretion (0.0067)	
	GnRH secretion (0.0172)		
		GnRH secretion (0.0356)	
MF—FDR	Histone modifying activity (0.0383)	Histone modifying activity (0.0004)	None
	Protein serine kinase activity (0.0166)	Histone H2AS1 kinase activity (0.0099)	
	Histone kinase activity (0.0166)	Histone kinase activity (0.0169)	
BP—FDR	**Cell morphogenesis (<0.0001)**	None	None
	Regulation of developmental growth (0.0001)		
	Cell junction assembly (0.0024)		

Pathways are separated into entries significant by FDR or *P* < .05. Only GO MF and BP termed are shown that meet significance by FDR. Up to 3 entries were chosen for each category based on (1) approximately 10% or higher representation in DEGs relative to total gene count in a network and (2) exclusion of immune-related processes. All significantly altered pathways and GO terms can be found in Supplemental File 3 (69). Bolded terms refer to highly significant entries (*P* < .0001).

A8535 exposure in pufferfish ovaries was moderately similar overall to PNX-20 and exhibited no highly significant pathway changes. Weaker changes were identified and some were similar to the PNX-20 treatment (Table 3). A8535 also induced small changes related to cortisol synthesis and secretion (*P* = .0067). Highly significant MF changes (14 total) were similar to PNX-20, with noted changes in histone-related functions (Table 3). No BPs were highly significant following A8535 treatment.

PT-91 exhibited the weakest effects, with no highly significant pathways. The only weaker pathway that was relatively robust and not directly related to immune function was associated with drug metabolism (*P* = .0183) (Table 3). PT-91 also had no significant MFs or BPs by FDR (Table 3).

### Comparisons across treatments within each species

**Figure 8:**
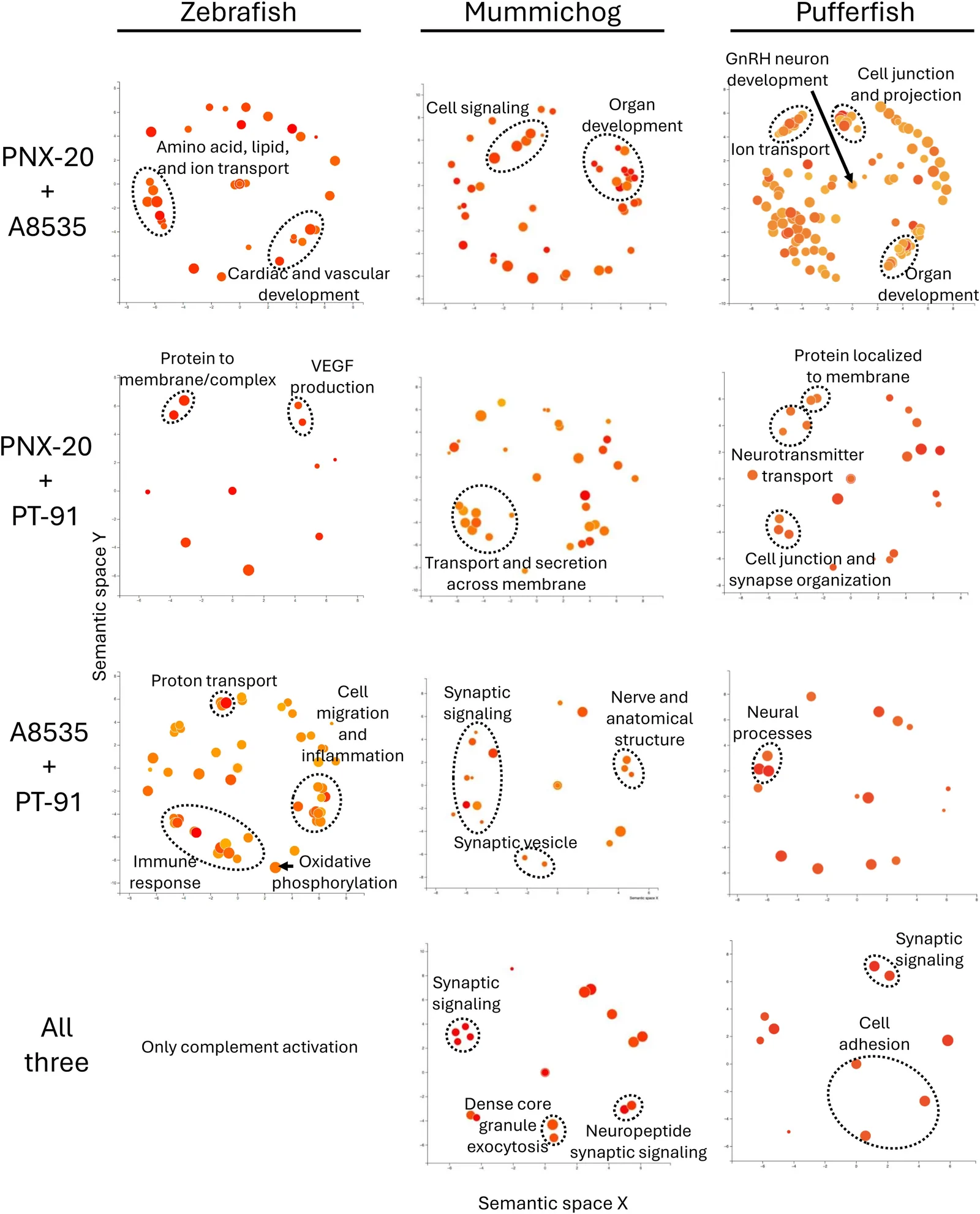
Significant (*P* < .05) GO BP terms found in multiple ovary treatments within a species, clustered into functional groupings using semantic similarity in REVIGO. Each row and column refers to a specific treatment combination and species, respectively. Darker and larger bubbles refer to terms with greater significance and more generality in the GO database, respectively. Selected BPs are also highlighted with arrows. Only one significant BP term was shared across all 3 treatments in zebrafish ovary (lower left). Complete lists for each species and treatment are available in species-specific meta-analysis BP tabs in Supplemental File 3 (69).

Zebrafish exhibited 11 pathways shared between A8535 and PT-91 treatments, while only 5 pathways were shared between PNX-20 and A8535 (see Supplemental File 3) (69). Many of these pathways in both comparisons were related to immune and metabolic functions. For instance, both PNX-20 and A8535 exhibited similar effects related to protein digestion and absorption (Table 4). A8535 and PT-91 treatments shared immune functions and changes in energy metabolism, including glucagon signaling, diabetic cardiomyopathy, and thermogenesis (Table 4). When all BPs (*P* < .05) were assessed across treatments, comparisons of PNX-20 + A8535, PNX-20 + PT-91, and A8535 + PT-91 exhibited 43, 13, and 104 shared terms, respectively. Using BP clustering, largely similar findings to previous analyses were evident in zebrafish, with PNX-20 + A8535 groups focused on metabolic processes (amino acid, lipid, and ion transport) and cardiac and vascular development (Fig. 8). PNX-20 + PT-91 shared only weak clustering related to membrane complexes and VEGF signaling. Oxidative phosphorylation was shared between A8535 and PT-91, along with several clusters related to proton transport, immune response, inflammation, and cell migration. Only a single BP (complement activation) was shared across all 3 treatments (Supplemental File 3) (69).

**Table 4:** Selected significantly altered gene pathways in zebrafish, mummichog, or pufferfish ovaries identified in multiple treatments (PNX-20 and A8535, PNX-20 and PT-91, A8535 and PT-91, or all 3)

Treatments	Species	Pathway	*P*-values in treatment order
PNX-20 + A8535	Zebrafish	Complement and coagulation cascades	.0486, .0249
		Protein digestion and absorption	.0071, .0025
		Systemic lupus erythematosus	.0243, .0009
	Mummichog	Pancreatic secretion	.0197, .0067
	Pufferfish	GnRH secretion	.0172, .0356
		GH synthesis, secretion, and action	.0070, .0016
		Type II diabetes mellitus	.0002, .0479
PNX-20 + PT-91	Zebrafish	None	—
	Mummichog	Neutrophil ECL trap formation	.0458, .0030
		Phototransduction	.0017, .0006
	Pufferfish	Dilated cardiomyopathy	.0025, .0016
		Hypertrophic cardiomyopathy	.0047, .0029
		Longevity regulating pathway	.0359, .0279
A8535 + PT-91	Zebrafish	Glucagon signaling pathway	.0113, .0137
		Diabetic cardiomyopathy	.0038, <.0001
		Thermogenesis	.0332, <.0001
	Mummichog	cAMP signaling pathway	.0009, .0394
	Pufferfish	Long-term potentiation	.0034, .0428
All 3 (PNX-20 + A8535 +PT-91)	Zebrafish	None	—
	Mummichog	None	—
	Pufferfish	Motor proteins	0.0422, 0.0336, 0.0078

Three pathways were chosen in each category that focused on metabolism or reproduction when able. *P*-values for each treatment are ordered as described in the Treatments column. All pathways in each category are listed under meta-analysis pathway tabs in Supplemental File 3 (69).

Mummichog exhibited only 4 total shared pathways between treatments (Table 4). Only pancreatic secretion was shared between PNX-20 and A8535, while only immune (neutrophil-related) and neural (phototransduction) pathways were shared between PNX-20 and PT-91. Only the cAMP signaling pathway was shared between A8535 and PT-91. Similarly to zebrafish, no pathways in mummichog were evident across all 3 treatments. Relatively more significant changes were identified using BPs, where comparisons of PNX-20 + A8535, PNX-20 + PT-91, and A8535 + PT-91 exhibited 54, 45, and 23 shared terms, respectively (see Supplemental File 3) (69). BP clustering was used to identify several groups within each comparison (Fig. 8). For example, PNX-20 + A8535 in mummichog exhibited clusters related to cell signaling and organ development, while the PNX-20 + PT-91 comparison was more scattered with only clustering evident for membrane functions. The agonist comparison (A8535 + PT-91) demonstrated consistent signals in synaptic structure. Similarly, all 3 treatments in mummichog shared clusters related to the synapse (Fig. 8).

In pufferfish, 9, 5, and 2 pathways were each shared between PNX-20 and A8535, PNX-20 and PT-91, and A8535 and PT-91, respectively (Supplemental File 3) (69). Both PNX-20 and A8535 exhibited pathways related to reproductive function (GnRH secretion) and metabolism (Table 4). In contrast, PNX-20 and PT-91 shared similarities in heart function and aging. A8535 and PT-91 shared a single pathway related to neural function. In addition, pufferfish was the only species to exhibit a pathway change in all 3 treatments, which was related to motor proteins. Pufferfish exhibited some of the greatest similarity in BPs across treatments, with PNX-20 + A8535, PNX-20 + PT-91, and A8535 + PT-91 comparisons exhibiting 232, 26, and 20 shared terms, respectively (see Supplemental File 3) (69). In BP clustering, ion transport, cell junction and projection, and organ development were major groups identified across PNX-20 + A8535 (Fig. 8). In contrast, PNX-20 + PT-91 exhibited clusters related to membrane proteins, neurotransmitter transport, and the synapse. A8535 + PT-91 shared relatively little in pufferfish, with only a small cluster related to neural functions. Similarly, across all 3 treatments, synaptic signaling was also clear with an additional cell adhesion group (Fig. 8).

### Comparisons across species

Some broad similarities across species were also evident in BP clusters (Fig. 8). For instance, across PNX-20 + A8535, BPs reflecting developmental processes were consistently identified in all species. Transport and protein localization to the membrane was also a consistent theme for PNX-20 + PT-91. In contrast, the combination of A8535 + PT-91 seemed to be more similar between mummichog and pufferfish (neural functions) than zebrafish (immune and energy production). This was also similar across all 3 treatments, where both mummichog and pufferfish, but not zebrafish, shared BPs associated with synaptic signaling.

**Figure 9:**
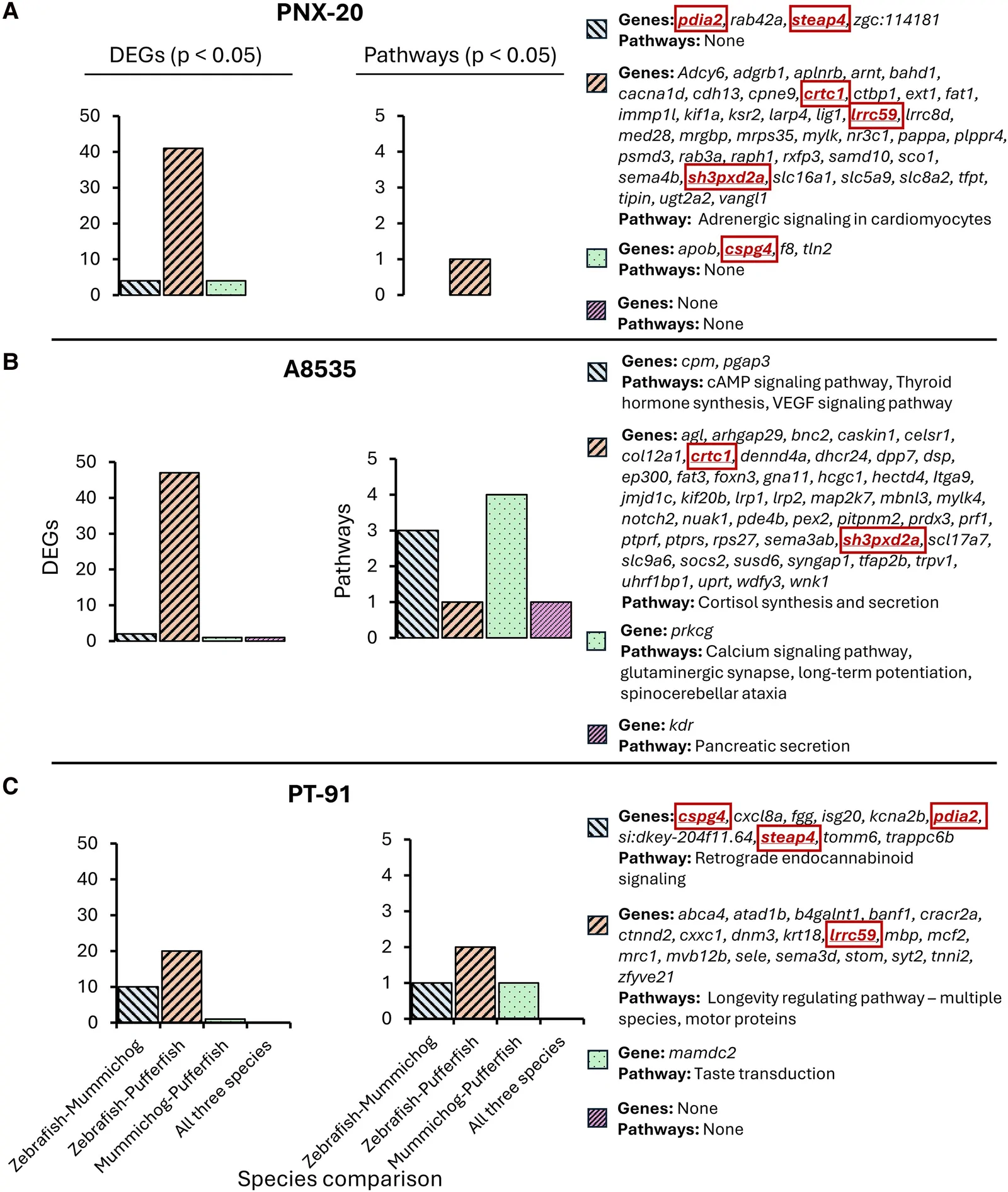
DEGs (left, *P* < .05) and pathways (right, *P* < .05) shared across multiple species within ovary treatments of PNX-20 (A), A8535 (B), and PT-91 (C). Categories (bottom axis) are separated into DEGs or pathways shared between zebrafish and mummichog, zebrafish and pufferfish, mummichog and pufferfish, and all 3 species. DEGs and pathways are listed for each category in legends to the right. Bolded, underlined, and boxed gene abbreviations refer to genes shared across both multiple species and treatments. These genes (6 total) are found in 2 ovary treatments among (A, B, and C).

Similarities across species were also evident in DEGs and broader pathways (Fig. 9). Zebrafish and pufferfish overall exhibited the greatest overlap in DEGs in all 3 treatments. In addition, several highly conserved patterns were identified across species (red blocks in Fig. 9). PNX-20 treatment exhibited 6 total genes (*crtc1*, *cspg4*, *lrrc59*, *pdia2*, *sh3pxd2a*, and *steap4*) that were both differentially expressed in multiple species and altered in an agonist treatment. Most of these changes were shared with PT-91 effects (*cspg4*, *lrrc59*, *pdia2*, and *steap4*) while fewer were shared with A8535 (*crtc1* and *sh3pxd2a*). No highly conserved changes were shared between the agonist treatments. Some broader gene patterns were evident as well. Both zebrafish and pufferfish exhibited significant changes in semaphorin family genes across all treatments (PNX-20 and *sema4b*, A8535 and *sema3ab*, PT-91 and *sema3d*), while myosin light chain kinase gene expression was altered in 2 treatments (PNX-20 and *mylk*, A8535 and *mylk4*). Finally, only one gene, a receptor for VEGF (*kdr*), was shared across all species following A8535 treatment.

Some similarities were also evident in pathways across species (Fig. 9). First, conserved PNX-20 changes were minimal, except for shared adrenergic signaling in cardiomyocytes in both zebrafish and pufferfish. Second, A8535 treatments exhibited the greatest similarities among the species. Zebrafish and mummichog shared cAMP signaling, thyroid hormone synthesis, and VEGF signaling. Zebrafish and pufferfish shared cortisol synthesis and secretion. Mummichog and pufferfish shared several likely neural-associated pathways. Third, PT-91 also induced some conserved pathway changes likely related to neural processes and motor proteins. Only A8535 exposure induced a shared pathway change in ovaries of all 3 species (pancreatic secretion).

### Confirmation of RNA-seq patterns using qPCR

Gene expression patterns in RNA-seq were largely confirmed using qPCR. Significant regressions (*P* < .05) between normalized RNA-seq counts and qPCR relative expression were evident in 4 of 6 selected zebrafish genes (Fig. S7) (50), 5 of 7 mummichog genes (Fig. S8) (50), and 5 of 7 pufferfish genes (Fig. S9) (50). Overall, ovarian patterns were more variable in qPCR than RNA-seq, and qPCR analyses did not exhibit significant changes across treatments at any gene. However, this was not unexpected and largely mirrored previous patterns observed in the fish ovary when exposed to PNX, which was likely caused by low sample sizes, small fold changes, and ovary-specific variability in expression (48). Broadly, genes that were confirmed by regression analyses also exhibited trends in expression that largely matched RNA-seq data.

### Steroid quantifications

Cortisol, 17-hydroxyprogesterone, testosterone, 11-KT, and (17)estradiol did not exhibit any significant difference in culture media compared to negative controls of any experimental treatments across all 3 species (Fig. S10) (50). Steroid data are provided in Supplemental File 4 (76).

## Discussion

Overall, novel agonist binding to fish SREB1s was supported. Our structural analyses revealed that the overall transmembrane architecture is highly conserved between human and fish SREB1 (RMSD <1.5 Å for all models). The dynamic behavior of the receptors did present unique computational challenges. Initial MD simulations showed high total-structure RMSD, which we identified as an artifact of inherent flexibility in the extended fifth and sixth transmembrane (TM5 and TM6) helices, as well as the loop regions. By applying TM-specific alignments, we demonstrated that the orthosteric binding pocket remains highly stable, providing a reliable environment for sustained ligand interaction.

A key finding of this study is the identification and structural comparison of the binding pocket hotspots. Previous studies on human SREB1 identified P170, L173, F186, and Y300 as critical residues for ligand binding (36). Our sequence, structural modeling, and alignments reveal an interesting evolutionary shift in the fish orthologs. While the hydrophobic and aromatic core is strongly conserved (human L173 to fish F176/175; human F186 to fish F189/188; human Y300 to fish Y302/301), the human proline 170 is replaced by glutamine (Q173/172) in all 3 fishes. This substitution from a rigid proline to a polar, flexible glutamine likely alters the local dynamics and electrostatic environment of the pocket. Despite this substitution, PRD confirmed that the conserved aromatic and hydrophobic residues, specifically Y302/301 and F78/77, serve as the primary energy drivers for both A8535 and PT-91, stabilizing the ligands via robust π-π stacking and van der Waals interactions.

The energetic profiling across both MM-GBSA and PMF further validates these agonists as potent SREB1 ligands in fish. While the *in silico* free energies quantify the thermodynamic stability of ligand binding, the transcriptomic results reflect downstream functional responses. Comparisons of these datasets reveal that, although related, binding affinity and biological activity are not directly proportional and are modulated by receptor-specific and species-dependent factors.

Ovary transcriptomic responses to the agonists were largely similar in scale to those induced by PNX, with some variations across species. Zebrafish responses followed expectations of agonist potencies, with the greatest gene and pathway changes following PT-91 exposure. Interestingly, the PMF profile for PT-91 binding in zebrafish produced the weakest BFE minimum among all ligand–receptor combinations, with no single dominant global minimum but instead multiple shallow local minima. This pattern suggests a highly dynamic binding mode, where the ligand is not tightly locked into a single conformation but instead transitions between multiple metastable states within the binding pocket. This behavior is consistent with the concept of functional selectivity (biased agonism), where ligands with lower binding affinity may still act as highly efficacious activators by promoting receptor conformational flexibility and facilitating transitions toward active signaling states. Despite having a divergent PMF profile, transcriptomic effects from PT-91 were still clearly more similar to the other agonist A8535 than PNX, which was expected based on their association to different receptors (33, 36). However, in the more derived fishes, mummichog and pufferfish, these patterns diverged likely due to species-specific characteristics, including receptor patterns and possible evolutionary differences in the SREB-activated response.

Mummichog exhibited the lowest response to endogenous PNX, and there was relatively little overlap in effects across any treatment or with other species. As a member of the Order Cyprinodontiformes, mummichog lost SREB3A and thus, may not exhibit similar PNX-induced effects to zebrafish (37). In addition, mummichog SREB1 exhibited somewhat reduced RMSD scores from HMs compared to the other species, and the PMF profiles for both agonists exhibited a single dominant minimum and lack of strong ligand-specific differentiation. Overall, reduced SREB1 effects may be characteristic of cyprinodontiformes and closely related species, since both the African turquoise killifish (*Nothobranchius furzeri*) and Japanese medaka (*Oryzias latipes*) have likely lost the SREB1 gene as well (37). While further research will be needed to confirm these patterns, gene expression changes from both SREB1- and SREB3-associated systems support a broadly depressed response in the mummichog ovary.

**Figure 10:**
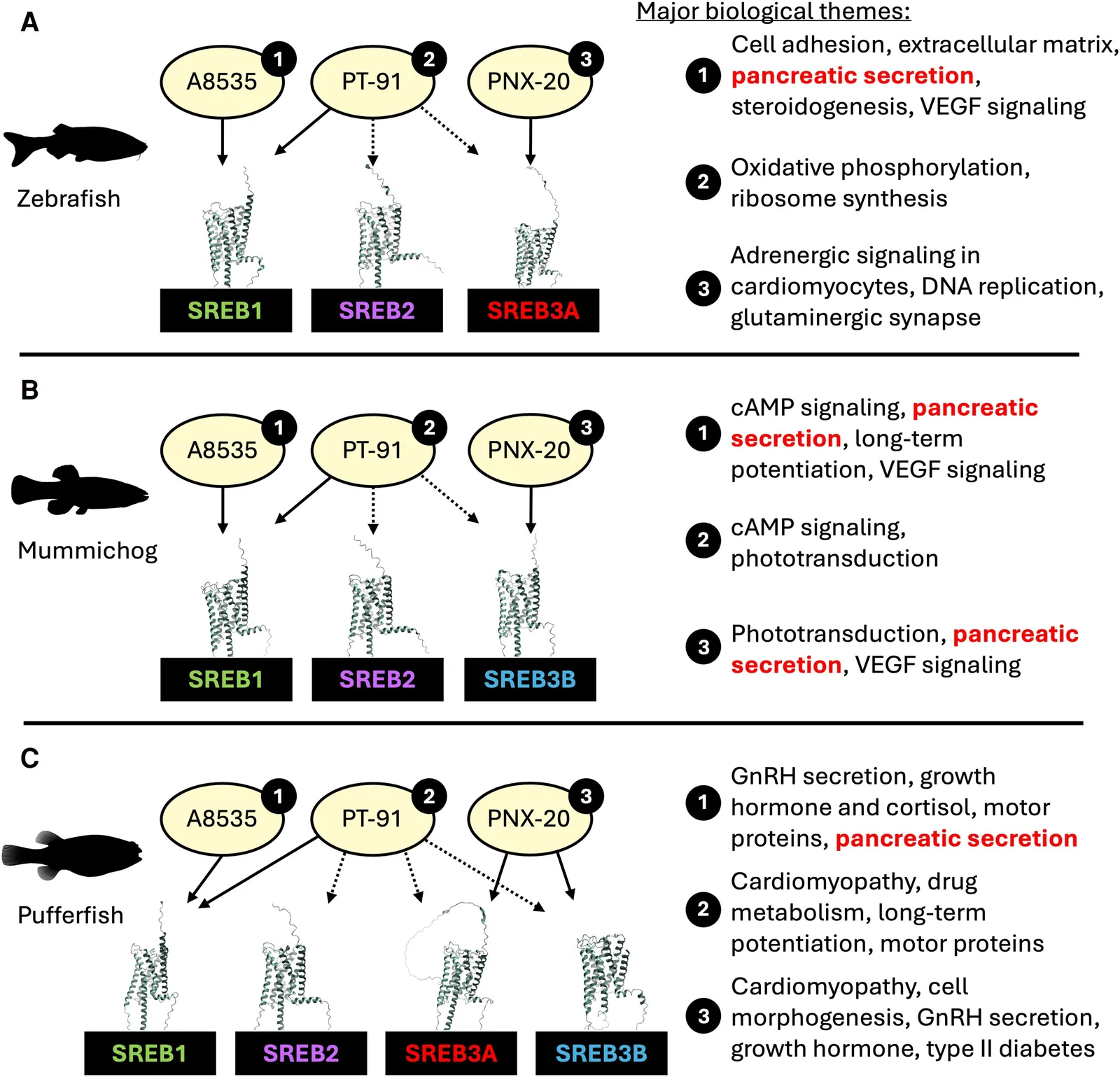
Summary of agonist (A8535 and PT-91) and PNX-20 associations with species-specific SREB receptors in zebrafish (*D. rerio*, A), mummichog (*F. heteroclitus*, B), and pufferfish (*D. nigroviridis*, C) with selected major biological themes (1, 2, and 3, respectively). Individual SREB protein models were used from AlphaFold or AlphaFold2 and imported into Flare (Cresset, England) for visualization (78-81). Solid lines from an agonist or ligand to a receptor indicate a known association, while dashed lines indicate potential but unconfirmed PT-91 effects on receptors beyond SREB1. Transcriptomic effects are summarized as themes, which refer to combinations of shortened pathway names or GO BP terms. Bold highlights refer to the only pathway altered across all 3 species.

Pufferfish exhibited a moderate response compared to other species. PNX and A8535 effects somewhat mirrored zebrafish, but with greater similarity between those 2 treatments, while PT-91-induced changes were severely reduced and more similar to mummichog results. However, unlike mummichog, pufferfish SREB1 molecular docking exhibited stronger probable PT-91 binding. In addition, SREB1 expression is both the greatest among these receptors in the pufferfish ovary and likely plays roles in ovarian development (39). Although variable species-specific effects in PT-91 binding requiring further validation cannot be discounted, this reduced pattern may also be due to a few factors involving the presence of both SREB3A and SREB3B. Preliminary research by our groups suggests additional PT-91 binding to other human SREBs, unlike SREB1-selective A8535 (data not shown), and this may also affect fish receptors as well. Indeed, 4 of the 7 amino acid positions predicted to be critical for PT-91 binding to pufferfish SREB1 are also present in the other SREBs (F78, Y302, L322, and V326). As a result, PT-91 effects may suggest a multi-SREB activation pattern. In pufferfish, this may trigger some opposing actions among the 2 SREB3s or lead to depressed responses by receptor desensitization when all 4 types are bound (40, 41, 77). Overall, some signal attenuation may be present in many of these agonist treatments, as all PMF profiles for SREB1 exhibited deep and well-defined free energy minima, except for zebrafish PT-91 that also exhibited the greatest transcriptomic response. Despite these patterns, many transcriptomic changes were still identified in the 3 species. A broad summary of species-specific responses is provided in Fig. 10.

All 3 fishes exhibited the most dramatic gene changes in energy metabolism and cell growth. The most significant signals were upregulation of transcription, translation, and oxidative phosphorylation in the zebrafish ovary following exposure to the potent PT-91 agonist, which were also observed to a lesser extent in A8535 and likely implicate SREB1 activation. Metabolic roles for SREB1 have been documented previously in several studies, including impacts on glycolysis, glucose metabolism, and insulin regulation in rodents (5, 7, 9, 45). Indeed, pancreatic-related gene changes may be highly conserved among SREB1 systems, as the pancreatic secretion pathway was altered across all 3 fish species in this study. Some similar metabolic effects are also likely shared with the SREB3 system, since PNX affects feeding behavior and is also a metabolic regulator in adipocytes and pancreatic islets (20, 25, 26). These similarities across receptors were reinforced in the present study, where PNX induced pathway changes in pancreatic secretion and type II diabetes in mummichog and pufferfish. Although these 2 species did not exhibit dramatic oxidative phosphorylation changes as in zebrafish, clear signals were instead identified in cell growth processes.

Broad changes in growth and development were consistent in all 3 species, especially when exposed to either PNX or A8535. Zebrafish ovaries in particular exhibited strong changes in cell adhesion and ECL matrix remodeling when treated with A8535, while weaker signals in mummichog and pufferfish centered on cell junctions, membrane organization, and development. While SREB roles in cell adhesion and growth are understudied, prior research supports functions in mammalian neurogenesis, and in fish these receptors are likely regulated by transcription factors that modulate cell proliferation and migration (10, 12, 38). Other growth-related processes such as vascular effects and angiogenesis were also shared. VEGF signaling was especially apparent in both SREB1 and SREB3-associated systems, and a VEGF receptor (kinase domain insert receptor, *kdr*) was the only single gene expression change detected in all 3 species. Other highly conserved changes included known drivers of cancer cell metabolism (*lrrc59*, *pdia2*, *steap4*), ECL matrix remodeling (*cspg4*, *sh3pxd2a*), and energy balance (*crtc1*) (82-87). SREB1 and SREB3 dysregulation are now being associated with ovarian and gastric cancers, respectively (88, 89), and all of these receptors likely regulate cell growth and proliferation across vertebrate systems.

Reproductive effects were overall weaker than anticipated, based on previous work in the vertebrate ovary that suggested SREB3 roles in folliculogenesis, steroidogenesis, and oocyte maturation (4, 46). While some increases were detected in zebrafish ovarian steroidogenesis via SREB1, other treatments and species exhibited little change, and no measured steroids were significantly altered. This is likely a product of both exposure time and ovary-specific effects. Germinal vesicle breakdown was observed in later ovarian stages in zebrafish following identical PNX treatment and 24 hours exposure, while the present study treated earlier stage follicles for only 6 hours (46). These short exposure times were highly effective in inducing *in vitro* reproductive effects in spotted scat (*Scatophagus argus*), but that research focused on hypothalamus and pituitary tissues and not ovaries (41, 44). Overall, ovarian reproductive effects of SREB system activation may manifest as downstream effects over longer time periods, since no significant changes in steroids were detected. More continuous or *in vivo* stimulation may be needed to induce reproductive changes in the ovary.

Greater immediate reproductive changes may occur upstream in the hypothalamic–pituitary–gonadal axis. This is also supported in the present study, where rather than steroidogenic changes, the GnRH secretion pathway was affected by both the SREB1 and SREB3 systems in the pufferfish ovary. Although this pathway was unaffected in zebrafish, this is not surprising given their lost GnRH functions (90, 91). Overall, the detection of neural signals in fish ovaries is not unusual and has been seen in treatments with spawning-inducing hormones or stage-specific functional changes (92-94). The link between SREB3 and GnRH secretion in the brain has been established through both PNX and GnRH (1-5) signaling, where these possible ligands regulate GnRH neuron functions (3, 12). This is the first study, however, to document changes in GnRH neuron-related expression following SREB1 activation in any vertebrate and suggests some redundancy across SREBs in reproductive function.

Neural signals in general were prominent in all 3 species, which highlight how SREBs can rapidly impact broad axonal and synaptic functions. Both zebrafish and pufferfish exhibited significant changes in the motor protein pathway across treatments as well as changes in several semaphorin family genes (*sema3ab*, *sema3d*, and *sema4b*) that likely guide axonal growth in nervous system development (95, 96). Even relatively weak changes across treatments in mummichog also implicated SREBs in synaptic structuring. While all SREBs are highly expressed in the vertebrate central nervous system in both mammals and fish, specific neural development roles have only been studied in a few mammalian systems, where SREB3 influences GnRH neuron migration and SREB2 impacts neurogenesis and brain size (2, 10, 11, 12, 37, 97). Although SREB2-specific effects cannot be identified in the present study, considerable functional overlap likely exists across all SREBs in nervous system development and embryonic growth. Indeed, similar spatial patterns for both *sreb2* and *sreb3a* transcripts are found in pufferfish oocytes that suggest these are stored maternal mRNAs for embryonic development (39). Overall, SREB systems seem to be strongly connected to neural development and neuron migration across vertebrates, with similar functions among family members that may result in little phenotypic change in single knockout experiments (10). As a result, more research across multiple SREB members, with multigene knockouts in CRISPR-Cas9 experiments, are needed to more fully decipher functions. While the present work provides a foundational framework for understanding some conserved SREB functions, there remains limited resolution at the individual receptor level. Further work on receptor-specific agonists and knockout models will be critical to more clearly identify each receptor's distinct role in metabolism and reproduction.

Our understanding of species-specific and conserved roles for SREBs across vertebrates remains incomplete. The evolutionary differences across fish in both receptor complements and effects highlight how these systems may exhibit divergent and lineage-specific functions despite being superconserved across many vertebrates in protein sequence (1). As such, while some effects are likely conserved, not all results from this study may translate to roles in other species. Indeed, prior PNX research highlights how comparative studies in these systems across both mammals and fish exhibit contradictory or context-specific effects in feed intake (26). More research will be needed with these agonists to identify which effects in fish are conserved with respect to mammalian systems. Additionally, the present study focused only on an *in vitro* ovarian tissue model, and *in vivo* approaches will be needed to more fully assess physiological and systemic effects. Coupled with the unclear status of SREB3 as a PNX receptor and the need for future agonist work, much uncertainty still remains about SREB functions. To overcome this, further research will be needed to clarify the association between PNX and SREB3, possibly through the identification of ligand binding to a receptor and whether SREB3 is a downstream mediator in a complex, integrative pathway. Synthetic agonist effects in mammalian systems will also need to be characterized, and the development of future SREB2- and SREB3-specific agonists will be particularly valuable for future transcriptomic and functional studies.

## Conclusion

Overall, PNX and the synthetic SREB1 agonists elicited overlapping ovarian transcriptional responses across fish species, supporting conserved vertebrate roles for SREBs in energy metabolism, cell proliferation, and developmental regulation. At the same time, clear species-specific differences were detected, most notably the stronger SREB1-associated responses linked to cell adhesion and mitochondrial function in zebrafish, and the comparatively muted response in mummichog, which may reflect evolutionary divergence in SREB signaling. By contrast, reproductive responses were limited under the present experimental conditions, as pathway-level effects were modest and no significant changes were detected in measured steroid levels. This suggests that longer exposure durations, *in vivo* paradigms, or upstream neuroendocrine activation may be required to reveal stronger reproductive effects.

Importantly, this study provides the first vertebrate evidence within a single experimental framework for potential functional overlap between SREB1- and SREB3-directed signaling. However, the individual contributions of each receptor remain unresolved, and further work will be needed to disentangle receptor-specific vs redundant functions and conserved or divergent functions in mammalian systems. In this regard, the development of SREB2-selective agonists, together with targeted multigene knockout approaches, will be particularly valuable. In parallel, our MD analyses support stable agonist binding to fish SREB1 orthologs, providing a pharmacological basis for the observed biological responses and a foundation for future functional assays. Finally, the marked interspecies variation observed here underscores the importance of comparative vertebrate models for resolving conserved and lineage-specific features of SREB biology.

## Data Availability

Original data generated and analyzed during this study are included in this published article or in the data repositories listed in References.
